# Comparative transcriptomics of stem bark reveals genes associated with bast fiber development in *Boehmeria nivea* L. gaud (ramie)

**DOI:** 10.1186/s12864-020-6457-8

**Published:** 2020-01-13

**Authors:** Jiyong Xie, Jiaqi Li, Yucheng Jie, Deyu Xie, Di Yang, Huazhong Shi, Yingli Zhong

**Affiliations:** 1 College of Bioscience and Biotechnology, Hunan Agricultural University, Changsha, 410128 China; 2 College of Agronomy, Hunan Agricultural University, Changsha, 410128 China; 3 Department of Plant and Microbial Biology, North Carolina State University, Raleigh, NC 27695 USA; 4 Department of Chemistry and Biochemistry, Texas Tech University, Lubbock, TX 79409 USA

**Keywords:** Ramie, Bast fiber, Phloem, Transcriptome

## Abstract

**Background:**

*Boehmeria nivea* L. Gaud (Ramie) produces one of the longest natural fibers in nature. The bark of ramie mainly comprises of the phloem tissue of stem and is the raw material for fiber. Therefore, identifying the molecular regulation of phloem development is important for understanding of bast fiber biosynthesis and improvement of fiber quality in ramie.

**Results:**

In this study, we collected top bud (TB), bark from internode elongating region (ER) and bark from internode fully elongated region (FER) from the ramie variety Zhongzhu No. 1. Histological study indicated that these samples contain phloem tissues at different developmental and maturation stages, with a higher degree of maturation of phloem tissue in FER. RNA sequencing (RNA-seq) was performed and de novo transcriptome was assembled. Unigenes and differentially expressed genes (DEGs) in these three samples were identified. The analysis of DEGs by using Gene Ontology (GO) and Kyoto Encyclopedia of Genes and Genomes (KEGG) revealed clear differences in gene expression between ER and FER. Some unigenes involved in secondary cell wall biosynthesis were up-regulated in both ER and FER, while unigenes for some cell wall components or cell wall modifications showed differential expression between ER and FER. In addition, the ethylene respond factors (ERFs) in the ethylene signaling pathway were up-regulated in FER, and *ent*-kaurenoic acid oxidase (KAO) and GA 20-oxidase (GA20ox) for gibberellins biosynthesis were up-regulated while GA 2-oxidase (GA2ox) for gibberellin inactivation was down-regulated in FER.

**Conclusions:**

Both morphological study and gene expression analysis supported a burst of phloem and vascular developmental processes during the fiber maturation in the ramie stem, and ethylene and gibberellin are likely to be involved in this process. Our findings provide novel insights into the phloem development and fiber maturation in ramie, which could be useful for fiber improvement in ramie and other fiber crops.

## Background

Natural plant fibers can be collected from the seeds of cotton, leaves of pina, fruits of coconut, stalk of bamboo, and bast of ramie (*Boehmeria nivea* L. Gaud). Among these fibers, ramie fiber is one of the longest and strongest natural fibers. Ramie produces fibers from its stem bark, which is originated from phloem tissue. Besides ramie, the well-known bast fiber crops include flax (*Linum usitatissimum*) and hemp (*Cannabis sativa*). By using genomic and transcriptomic analysis, significant progress has been made on bast fiber study in flax, hemp and ramie in recent years [1–7]. Through transcriptomic profiling, several secondary cell wall synthesis related proteins such as cellulose synthase, expansin and xyloglucan endotransglucosylase/hydrolase (XTH) were identified to be likely involved in fiber development in ramie [7]. In addition, enhanced gibberellin biosynthesis and Walls Are Thin1 (WAT1) related proteins might be important in domestication process of ramie varieties [4, 6]. Ramie has a vigorous vegetative growth, and its stem undergoes obvious elongation and thickening processes. Both ramie and flax initiate and produce primary phloem fibers in stem from the shoot apical meristem (SAM) [1]. Ramie also produces secondary phloem fibers in stem, which is similar to hemp or tension wood of poplar (*Populus tremula*). The secondary phloem is originated from the vascular cambium, which is a typical process in dicotyledonous plants with secondary stem thickening [8, 9].

Although the developmental process of fiber in ramie still requires detailed study, the production of both primary and secondary phloem fibers is believed to depend on secondary cell wall synthesis. The studies on the compositions of secondary cell wall of fiber cells, i.e. the proportion of cellulose, hemicelluloses and lignin, indicated that these cell wall components vary among different fiber plants [10], and the cell wall components even differ in the same cell type in different parts of the model plant Arabidopsis [11–13]. In fact, secondary cell wall formation is a complex process involving signaling events leading to transcriptional activation of secondary cell wall related genes, which results in the biosynthesis and assembly of secondary cell wall. Gene transcriptional regulatory networks and signaling cascade integrating signals for secondary cell wall biosynthesis have been gradually uncovered in recent years. NAC (NO APICAL MERISTEM, ATAF1, ATAF2, and CUP-SHAPED COTYLEDON 2) and MYB (myeloblastosis) transcription factors are thought to be the master switches that regulate the downstream transcription factors involved in the weaving of the network [14–18]. In one of the proposed models, at least three layers of regulators, which include NAC domain master regulators in the tier 3, two MYB domain regulators in the tier 2 and many other regulators in the tier 1, are likely to be directly involved in regulating secondary cell wall biosynthesis [18].

Secondary cell wall formation is regulated by phytohormones including auxin, ethylene and gibberellin (GA) [19, 20]. Auxin is a well-known hormone crucial for plant cell wall development [21]. In addition, ethylene signaling has recently been recognized to be necessary for the deposition of gelatinous layer of fiber cells [22, 23]. Ethylene signaling involves the perception of the hormone by the ER-localized receptor, and upon ethylene binding, the negative regulator CTR1 is released from the receptor, resulting in non-phosphorylation of the ER-localized EIN2. The C-terminus of the unphosphorylated EIN2 is cleaved and moved to the nucleus and thus stabilizes EIN3/EIL1, which activates the transcription of ERFs leading to the induction or repression of the downstream ethylene responsive genes [24]. GA is also implicated in secondary cell wall and fiber development [25, 26]. In the GA biosynthetic pathway, ent-kaurene oxidase (KO) and ent-kaurenoic acid oxidase (KAO) act in the early steps to produce GA12, which is subsequently converted to active forms of GA by two crucial enzymes, GA 20-oxidase (GA20ox) and GA 3-oxidase (GA3ox). In the GA catabolic pathway, GA 2-oxidase (GA2ox) converts active GAs to inactive forms [27].

Fiber development in ramie stem is a continuous and systematic process along the stem tissue, and there is no clear “snap point” to mark the transition from elongation to fiber thickening, which is a process resulting in changes in fiber mechanical properties [28]. In an effort of identifying the molecular regulation of phloem fiber cell development, we adopted a method to collect samples with different degrees of phloem maturation from the shoot of ramie. In this study, we collected the phloem tissues at different developmental stages from three regions of the stem: top bud (TB), internode elongating region (ER) and internode fully elongated region (FER). We performed RNA-seq and analyzed the gene expression profiles of these three tissues. Our results revealed key genes and pathways that are possibly responsible for the distinct secondary phloem formation and fiber development in ramie.

## Results

### Different segments of stem bark exhibit distinct morphological features

**Fig. 1:**
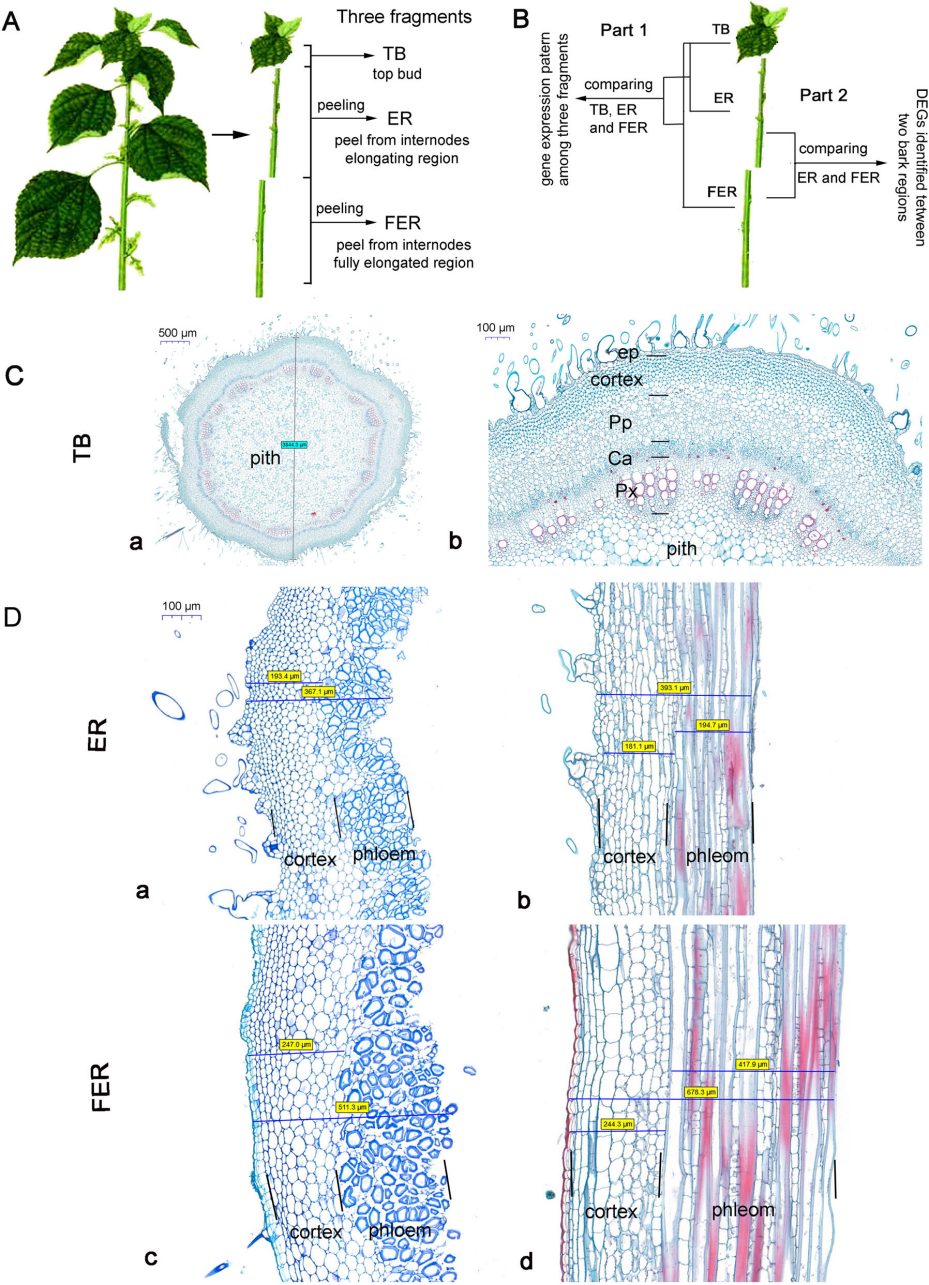
Ramie materials and transcriptome comparison strategy among samples. **a** Truncation of ramie shoots. The shoots were cut into three sections including top bud, elongating region (ER) stem and fully elongated region (FER) stem. The leaves were removed, and the ER and FER samples were collected by peeling the bark from the central woody column of the stem. **b** The strategy of DEG identification by comparing the transcriptomes between different samples. **c** Cross section of TB with 2 times magnification (a), 10 times (b). Scale bars were indicated respectively. ep: epidermal layer; Pp: primary phloem; Ca: cambia; Px: primary xylem. **d** Cross and lengthwise sections of ER (a and b) and FER (c and d) samples magnified 40 times. Scale bar represented 100 μm and is the same for **a**, **b**, **c** and **d**

Ramie fibers continuously develop along the stem during plant’s growth, while the internodes of the stem show obvious elongation only until the plant is fully elongated. To analyze the developmental stages of fiber formation and the gene expression profiles, three parts of ramie’s shoot were harvested, including top bud (TB), internode elongating region (ER) of stem and internode fully elongated region (FER) of stem (Fig.1a). The top buds and the barks peeled off from both ER and FER regions were used for histological analysis and RNA extraction. The scheme for RNA-seq data analysis is illustrated in Fig. 1b. The cross and longitudinal sections of TB, ER and FER were analyzed (Fig. 1c and d). In the TB sample, ramie has amphicribral vascular bundle, which is different from flax or hemp plants but is similar to woody plant with continuous cambia within and outside the vascular bundles (Fig. 1c). The vascular structure in TB is characteristic of multiple layers of primary phloem without obvious boundary between vascular bundles. In ER and FER, clear differences were observed between these two regions (Fig. 1d). Firstly, FER has thicker bark than ER, and FER barks consist of more enlarged cells and more layers of phloem tissues. Secondly, fiber cells show thicker cell wall in FER without an increase in cell size; the thickness of the fiber cell wall is about 5.38 μm in FER vs. 1.87 μm in ER (Additional file 1: Table S1). Thirdly, the cell wall of the fibers from FER phloem contains more lignin than that from ER, which is indicated by stronger red color of safranin dye staining (Fig. 1D-b and 1D-d). The differences among these three samples indicate different developing stages of phloem fiber cells. Therefore, we used these samples for gene expression profiling attempting to identify genes important for fiber development in ramie.

### Assembly of de novo transcriptome and identification of unigenes

**Fig. 2:**
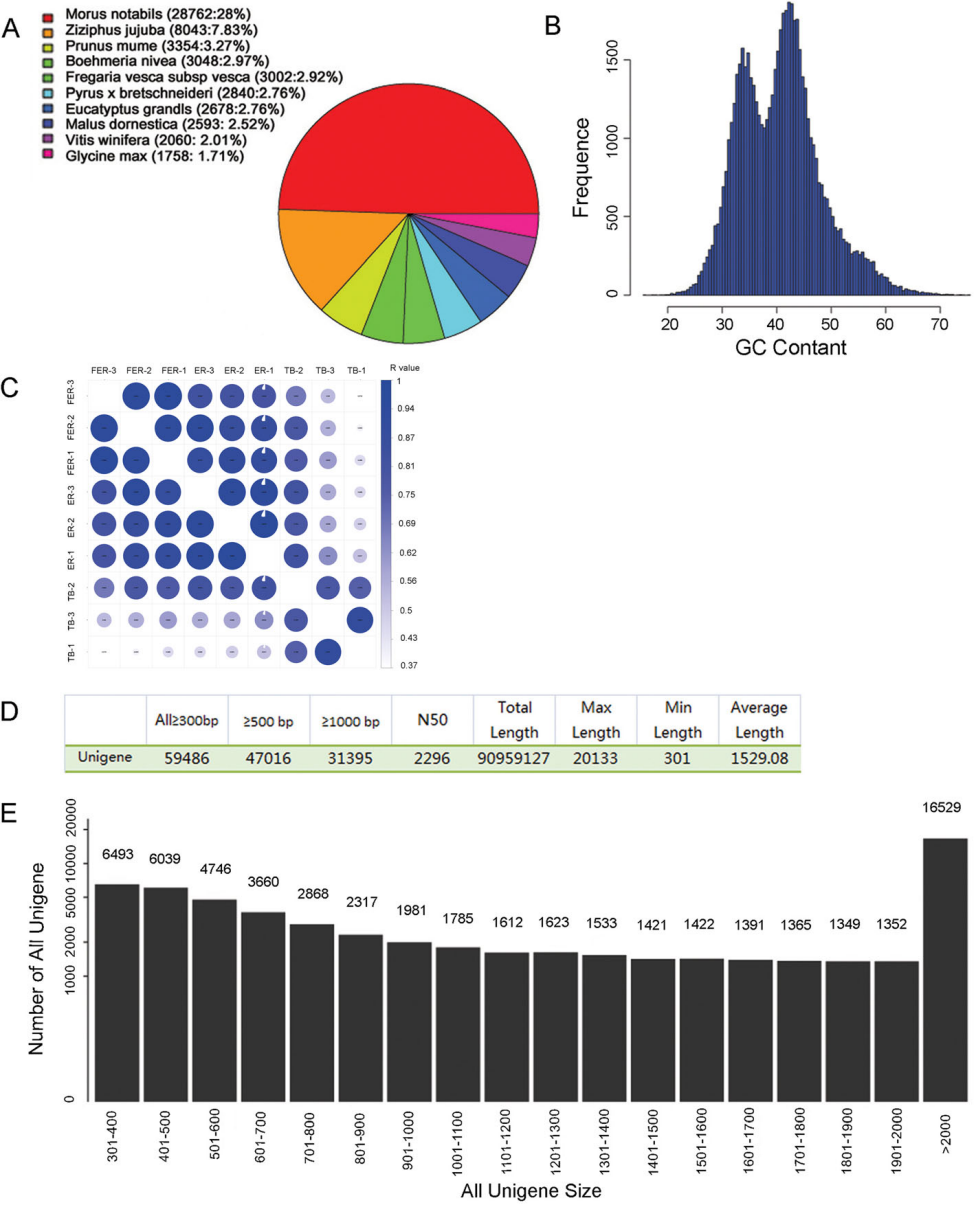
Characterization of the transcriptome and assembled unigenes in ramie. **a** 10 species with the most matching reads to our data. Different colors represent different species, and the area was corresponding to the quantity of matching reads in the organism. The amount and the percentage of matching reads were indicated in the brackets. **b** GC content distribution of all unigenes. **c** Sample to sample relationship matrix. **d** Statistics of unigene length. **e** Size distribution of all assembled unigenes

Thirty-three RNA samples were collected and subjected to the next generation sequencing (NGS), and the RNA-seq data, including the 9 submitted SRA files (SRR9112644-SRR9112651), were analyzed. More than 5G sequences with clean bases from each sample was obtained, and thus the total analyzed clean bases were about 1.7E^+ 11^. The genome size of Zhongzhu No. 1 is approximately 340 Mb [3, 4]. Therefore, the depth of the RNA-seq data used in this study is sufficient for a high quality de novo assembly of transcriptome for the expressed genes from the top bud and stem bark tissues. The 10 species with the most matching reads to our RNA-seq data were shown in Fig. 2a. Among all the reads generated, 3048 reads match with those in *Boehmeria nivea*, and the highest matching ratio (28%) was found to be with *Morus notabils*. Overall, there were 59,486 unigenes assembled with the length longer than 300 bp, 47,016 unigenes longer than 500 bp, and 31,395 unigenes longer than 1000 bp. The GC content distribution of all unigenes was shown in Fig. 2b, and two peaks appeared between the range of 30 and 45%. The correlation analysis showed that the three replicates of each sample were closely correlated (Fig. 2c). The detailed size distribution of all unigenes was illustrated in Fig. 2d and e. The sequence of each unigene was subsequently processed by blast to NR, SWISSPROT and KOG databases, respectively, and the annotations were obtained according to the most similar protein or gene with e < 1e^− 5^.

### Identification of differentially expressed genes (DEGs) and expression patterns among TB, ER and FER

**Fig. 3:**
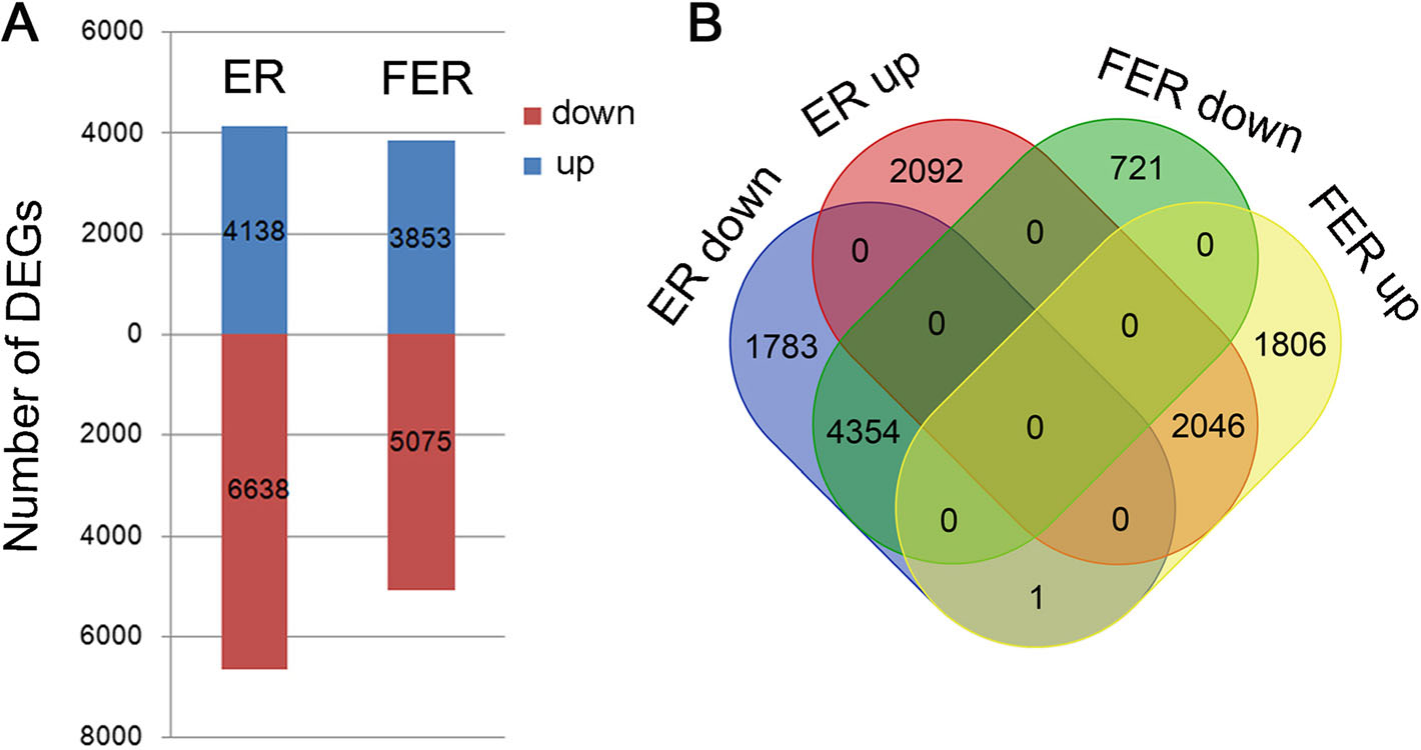
Differentially expressed genes between ER or FER stem section and top bud. **a** The number of DEGs identified by comparing ER or FER with TB. DEGs of ≥2 fold changes with *P*-value less than 0.05 were included. **b** VEN diagrams of DEGs in ER and FER comparing with TB

**Fig. 4:**
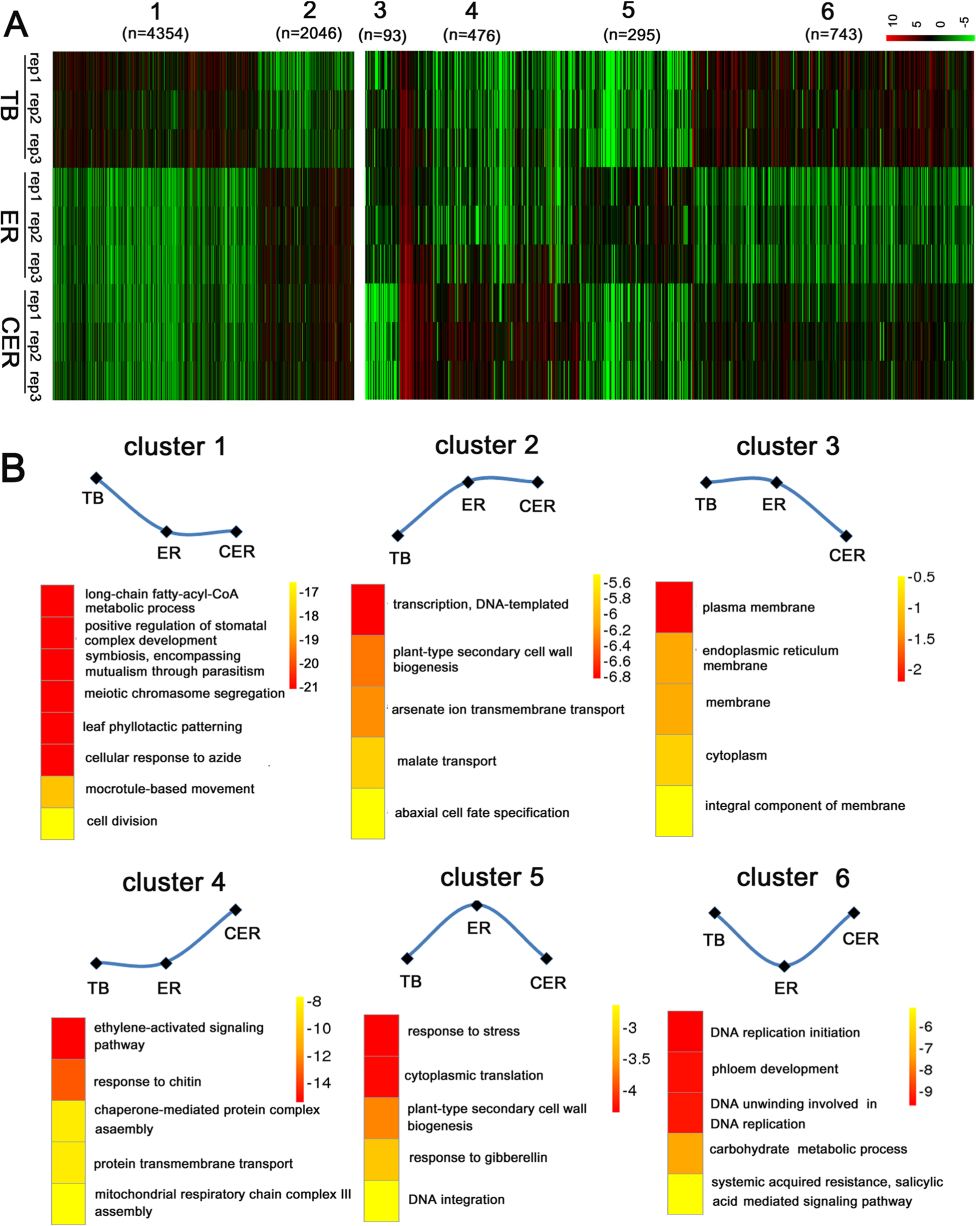
Gene expression patterns among TB, ER and FER regions. **a** The heatmaps of six clusters of DEGs among TB, ER and FER. The red color indicates upregulation, while green color represents downregulation. **b** Schematic curves for gene expression patterns among TB, ER and CER, and GO analysis of DEGs in each cluster

DEGs among the three tissues were identified following the scheme shown in Fig. 1b. When compared with TB, there were 4138 unigenes up-regulated and 6638 unigenes down-regulated in the ER, and 3853 unigenes up-regulated and 5075 unigenes down-regulated in the FER (Fig. 3a). The VENN diagram showed that the DEGs among these 3 samples were grouped in 6 distinct clusters (Fig. 3b). The heatmaps of the expression of these clustered genes were shown in Fig. 4a, and the schematic map of the expression patterns and GO analysis were illustrated in Fig. 4b.

The cluster 1 and 2 contain the most DEGs with 4354 up- and 2046 down-regulated unigenes only in TB (Fig. 4a and b). The cluster 1 DEGs consist of the unigenes with higher expression level in TB but lower expression level in both bark regions. GO analysis showed that these DEGs are involved in meiotic chromosome segregation and cell division and stomatal or leaf development (Fig.4b). GO analysis of the DEGs in cluster 2 showed that up-regulated transcription factors or transcription processes and the plant-type secondary cell wall biogenesis are among the top categories. Fifty-five unigene contigs for cell wall components or cell wall biogenesis and modification related factors were identified in the cluster 2 (Additional file 1: Table S2). These factors include Cellulose Synthase A Catalytic Subunit 3 and 8 (CesA 3 and 8), Fasciclin-like Arabinogalactan Protein (FLA), beta-galactosidase (BGAL), several pectinesterase/pectinesterase inhibitors (PMEs/PMEIs) and the enzymes for the synthesis of other cell wall components, such as glucuronoxylan glucuronosyltransferase, galacturonosyltransferase, endochitinase, callose synthase, xyloglucan glycosyltransferase, XTHs, etc. (Additional file 1: Table S2).

The cluster 3 and 4 show the unigenes up- or down-regulated only in FER (Fig. 4a and b). In these two clusters, there were 93 unigenes down-regulated and 476 unigenes up-regulated only in the FER. In cluster 3, a small amount of unigenes for membrane construction were down-regulated in FER. In cluster 4, relatively more unigenes were up-regulated in FER comparing with the down-regulated unigenes in cluster 3. Among these up-regulated unigenes, ethylene signaling pathway genes were the most enriched unigenes. There were totally 39 transcription factors up-regulated only in FER, and 18 out of the 39 were ethylene activating unigenes (Additional file 1: Table S3). The DEGs only in the ER were clustered in cluster 5 and 6. Interestingly, some phloem development related unigenes were found to be down-regulated only in ER when compared with those in both TB and CER (Fig.4b).

In addition to the expression patterns analyzed among TB, ER and FER, DEGs between TB and ER or FER were also analyzed and GO analyses were performed. The top 10 items of three GO terms were shown in Additional file 2: Figures S1 and S2. When compared with TB sample, the barks of FER showed gene expression patterns distinct from the barks of ER.

### GO analysis of DEGs between ER and FER

**Fig. 5:**
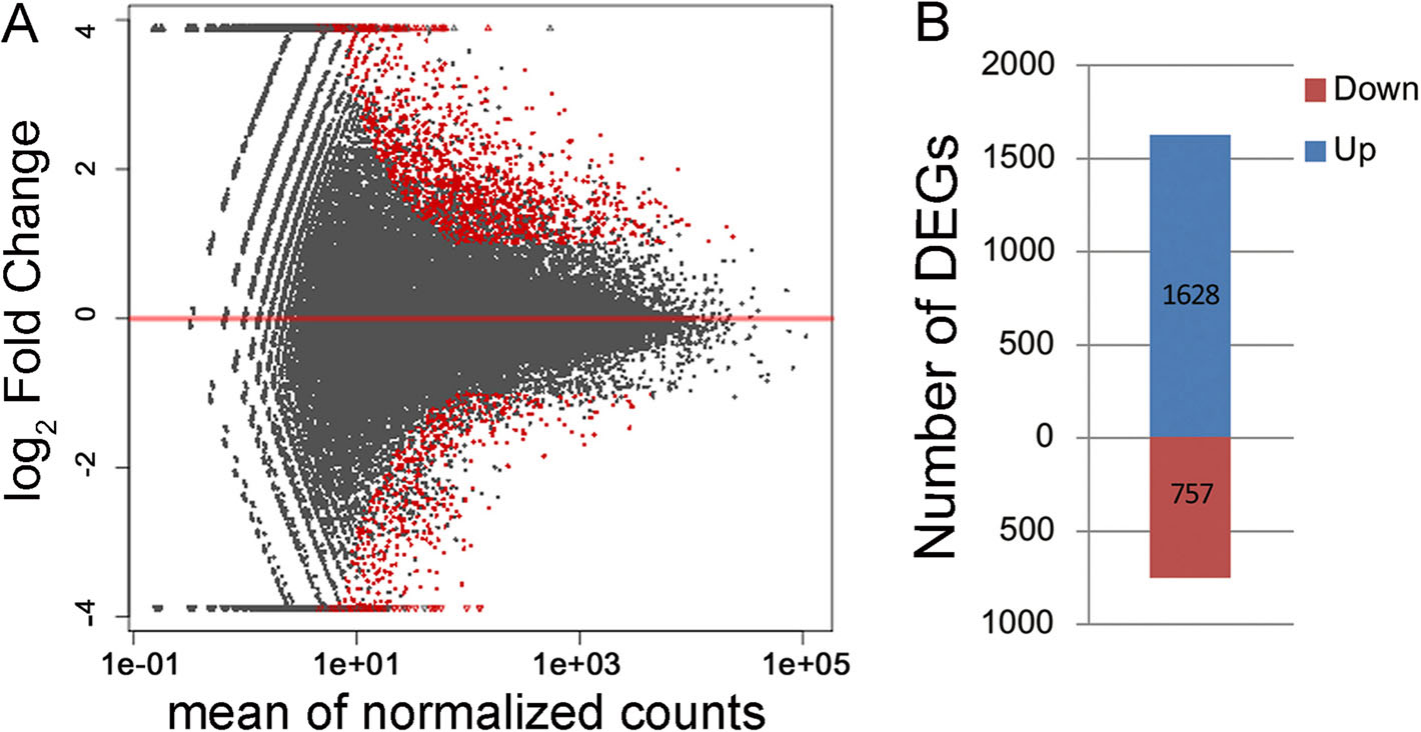
The DEGs between FER with ER. **a** Fold change distribution of DEGs of ER vs. FER. The X axis is the normalized average expression value of all identified unigenes, and the Y axis is log_2_Fold Changes. The red color indicates significant DEGs with more than 2 fold changes. **b** The DEG number of FER vs. ER. The number of up-regulated DEGs in FER was 1628, while the number of down-regulated DEGs was 757

**Fig. 6:**
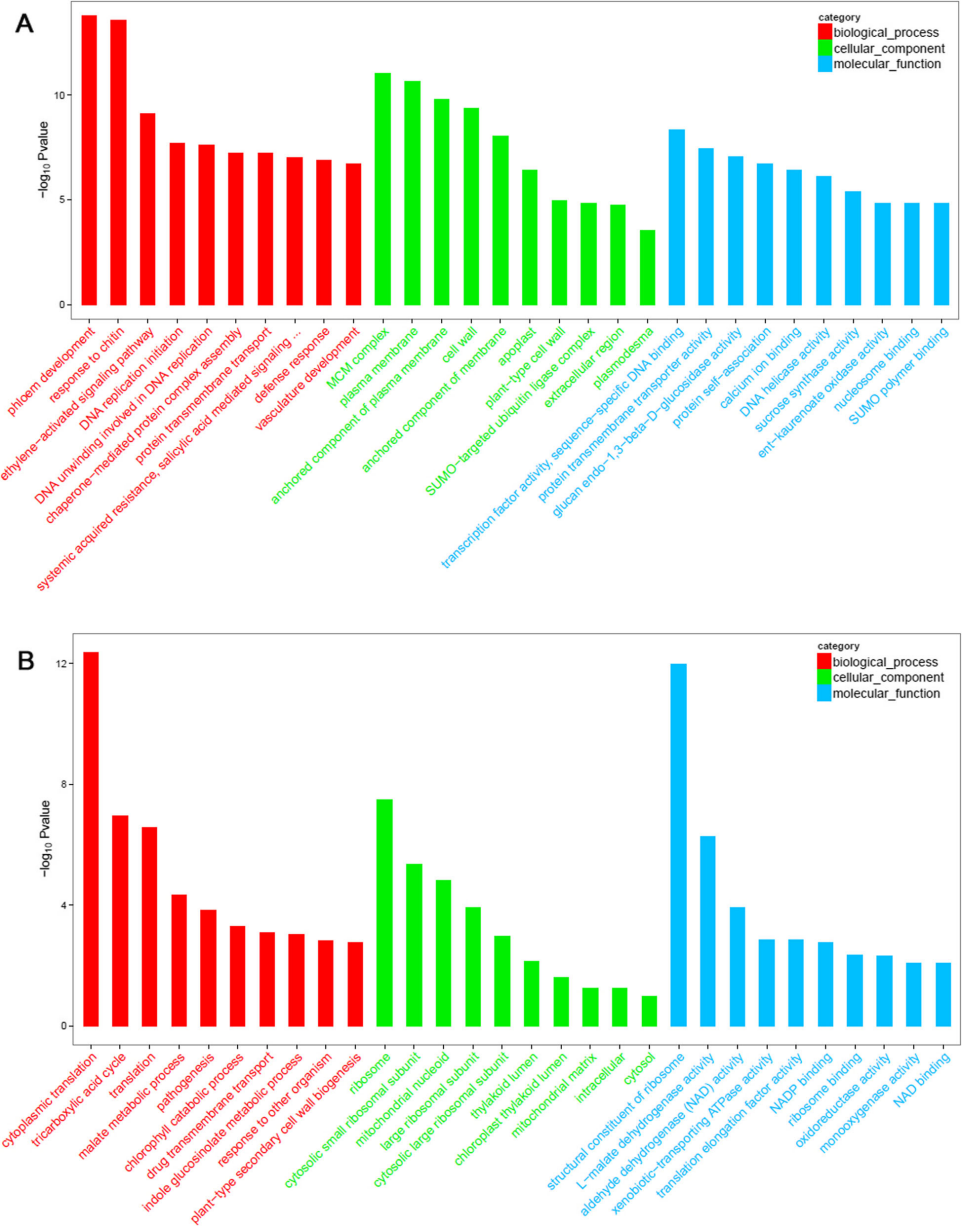
GO analysis of the DEGs between FER and ER bark of ramie. **a** GO analysis of the up-regulated genes in FER comparing with ER. **b** GO analysis of the down-regulated genes in FER comparing with ER. Top ten items were presented. Different colors represent different GO terms, e.g. Red for biological process, green for cellular component and blue for molecular function

There were 1628 up-regulated unigenes and 757 down-regulated unigenes identified in ramie’s bark of FER when compared with ER (Fig. 5). GO analysis shown in Fig. 6 revealed the top 10 up-regulated biological processes including phloem development, response to chitin, ethylene-activated signaling pathway, DNA replication, salicylic acid mediated signaling, defense response, protein transmembrane transport and vasculature development, and the top 10 down-regulated biological processes including cytoplasmic translation, tricarboxylic acid cycle, indole glucosinolate metabolic process, plant-type secondary cell wall biogenesis, etc.. The up-regulated genes in the activation of ethylene signaling pathway in FER is listed in Additional file 1: Table S4. Overall 21 unigenes or contigs of 14 *Ethylene Respond Factors* (*ERFs*) were up-regulated in FER, which include *ERF1*, *ERF1A*, *ERF1B*, *ERF2*, *ERF3*, *ERF5*, *ERF17*, *ERF22*, *ERF53*, *ERF61*, *ERF71*, *ERF109*, *PAR2–13* and *PAP2–4* (Additional file 1: Table S5).

### KEGG analysis of DEGs between ER and FER

**Fig. 7:**
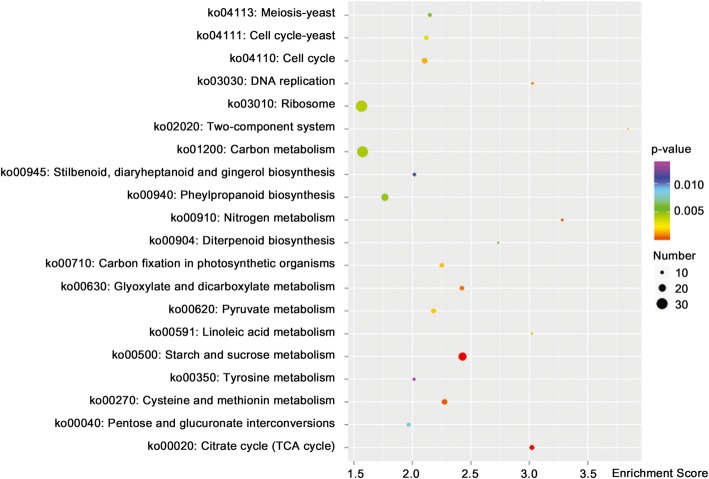
KEGG enrichment of the DEGs between FER and ER. Top 20 categories are shown. The X axis is enrichment score, and the areas of the bubbles indicate the DEG numbers, and the color variation of the bubbles from purple to red indicates decreasing *P* value

The KEGG analysis of total DEGs from FER vs. ER revealed additional information to the GO analysis. The KEGG analysis indicated that these DEGs are involved in the pathways of starch and sucrose metabolism, citrate cycle, nitrogen metabolism, cysteine and methionine metabolism, ribosome, diterpenoid biosynthesis, phenylpropanoid biosynthesis, DNA replication, cell cycle, etc. (Fig. 7 and Additional file 1: Table S7).

**Fig. 8:**
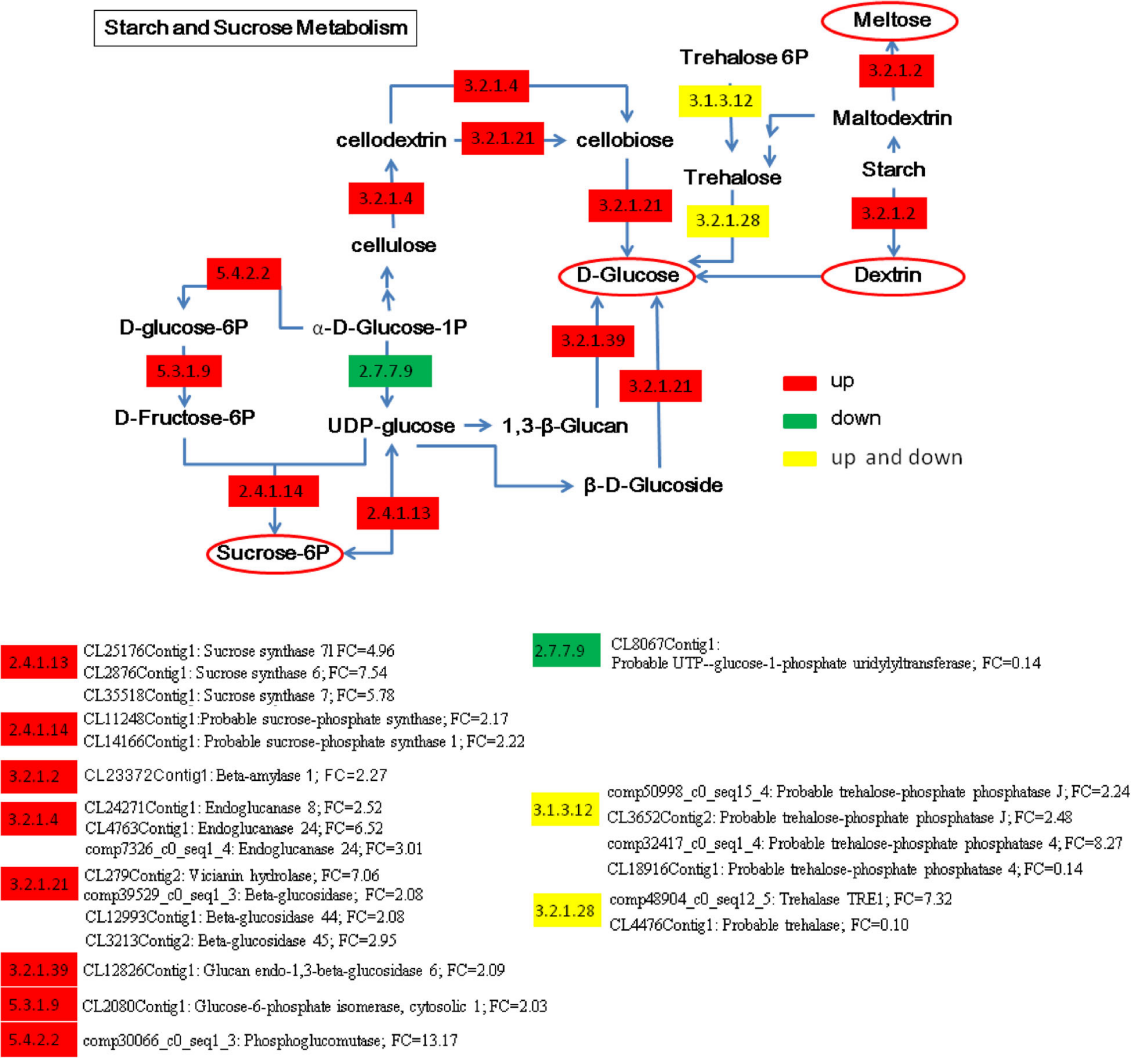
Different regulation in the starch and sucrose metabolism pathways between elongating and fully elongated regions of ramie stem bark. Multiple processes of free D-Glucose production might be enhanced in FER. Other sugar production processes through sucrose-6P, maltose and dextrin might also e increased in FER

From the KEGG analysis, we found that the expression of 23 unigenes encoding 11 enzymes in the starch and sucrose metabolisms differed between ER and FER. These enzymes include sucrose synthase (EC2.4.1.13), sucrose-phosphate synthase (EC2.4.1.14), bata-amylase EC3.2.1.2, endoglucanase (EC3.2.1.4), bata-glucosidase (EC3.2.1.21), glucan endo-1, 3-beta-glucosidase (EC3.2.1.39), glucose-6-phosphate isomerase (EC5.3.1.9), phosphoglucomutase (EC5.4.2.2), UTP-glucose-1-phosphate uridylyltransferase (EC2.7.7.9), trehalose phosphatase (EC3.1.3.12) and trehalase (EC3.2.1.28) (Fig. 8). Most of these enzyme-encoding unigenes were up-regulated in FER, which suggests that multiple pathways for free D-glucose production might be enhanced in FER. In addition, other sugar producing processes such as sucrose-6P, maltose and dextrin might also be enhanced in FER. The increase in these sugar precursors could be important in providing building materials for the secondary cell wall biogenesis in ramie.

**Fig. 9:**
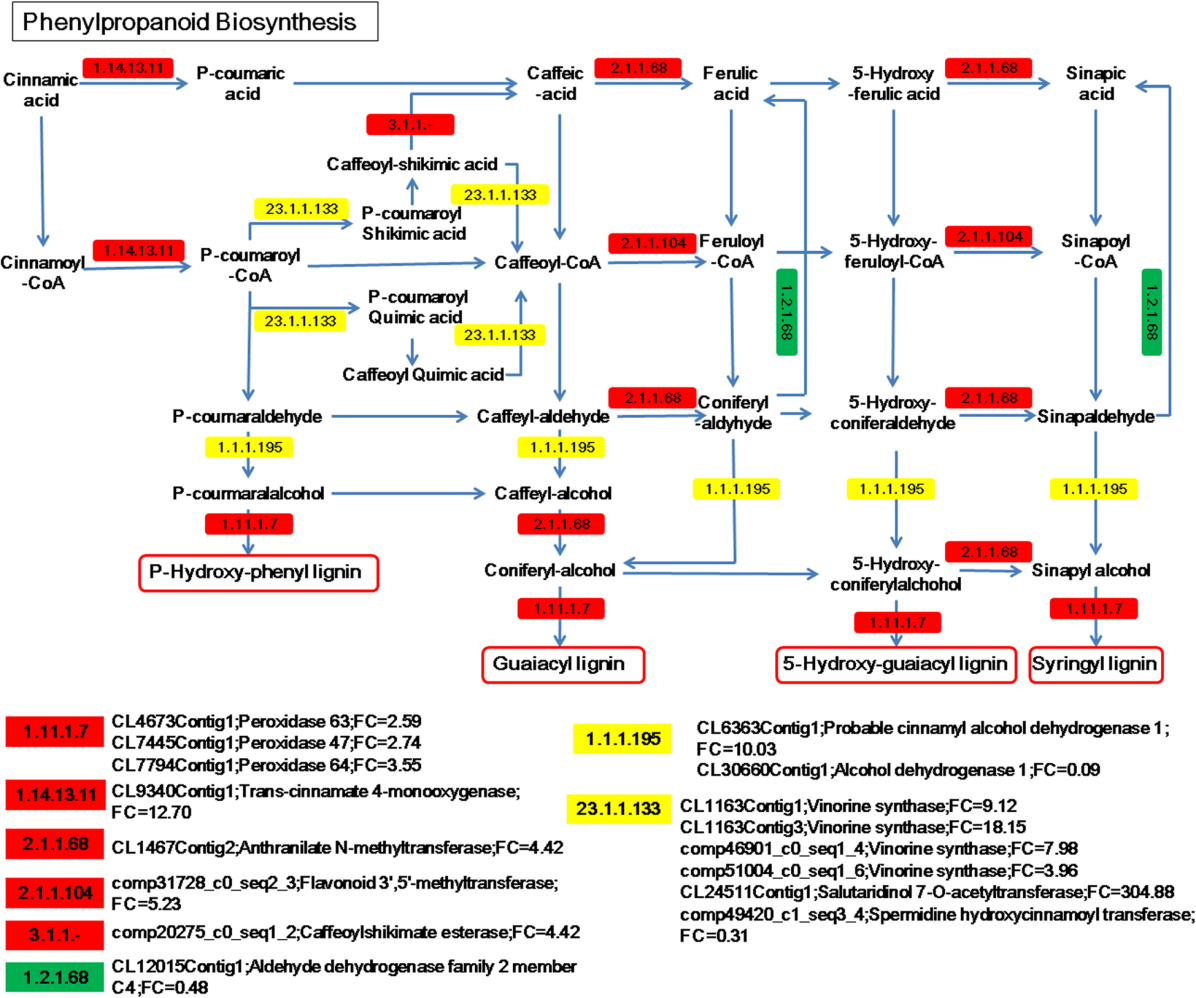
Up-regulation of lignin synthesis related genes of the phenylpropanoid biosynthesis pathways in FER. The unigenes encoding lignin synthesis enzymes including P-hydroxy-phenyl lignin, guaiacyl lignin, 5-Hydroxy-guaiacyl lignin and syringyl lignin were all up-regulated in FER. Red color stands for up-regulation, green color represents down-regulation and yellow means up or down regulation

More lignin accumulation in FER was observed by the staining with safranin dye (Fig. 1D-d), and KEGG analysis identified the up-regulation of enzymes responsible for phenylpropanoid biosynthesis in this region (Figs. 7 and 9). In the pheylpropanoid biosynthesis pathway (KO00940), 16 up-regulated unigenes encode enzymes including peroxidase (EC1.11.1.7), trans-cinnamate 4-monooxygenase (EC1.14.13.11), anthranilate N-methyltransferase (EC2.1.1.68), flavonoid 3′,5′-methyltransferase (EC2.1.1.104), beta-glucosidase (EC3.2.1.21), caffeylshikimate esterase and vinorine synthase, some of which were among the DEGs contributing to secondary cell wall synthesis (Fig. 9 and Additional file 1: Table S7). Most enzyme-encoding genes involved in the biosynthesis of lignin were up-regulated in FER, and up-regulation of several PMEs in FER was also identified (Additional file 1: Table S7).

**Fig. 10:**
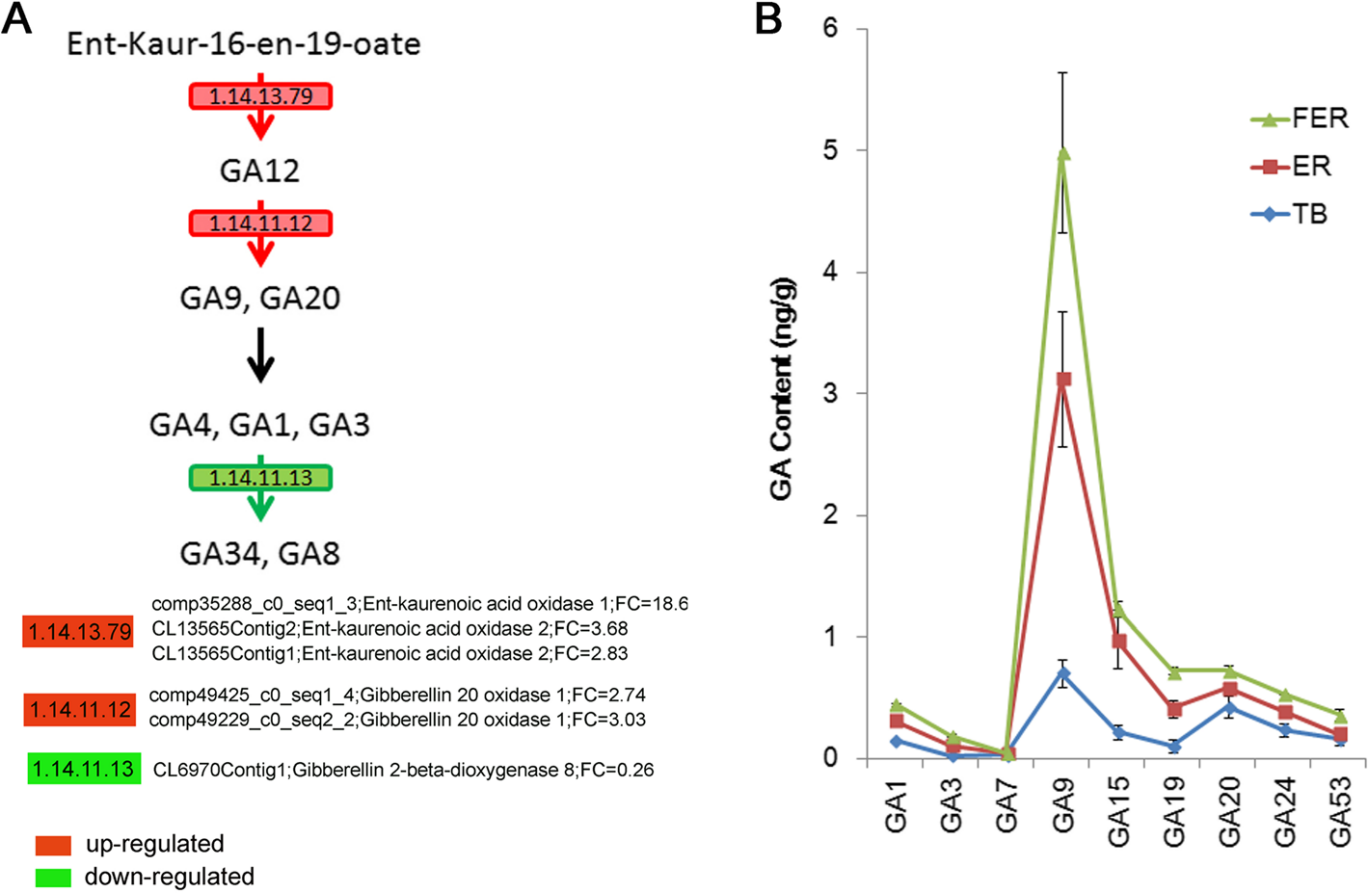
Altered expression of the key genes in the GA metabolism pathway and detection of GA contents. **a** The unigenes encoding enzymes of Ent-kaurenoic acid oxidase (EC.1.14.13.79) and Gibberellin 20 oxidase (EC.1.14.11.12) were up-regulated in FER. And gibberellins 2-beta-dioxygenase (EC.1.14.11.13) was down-regulated in FER. **b** Detection of GAs in different ramie sections. Nine types of GAs were shown in the assays

Interestingly, in the diterpenoid biosynthesis pathway, unigenes encoding enzymes such as Ent-kaurenoic acid oxidase 2 (KAO2) (EC1.14.13.79) and gibberellins 20 oxidase (GA20ox) (EC1.14.11.12) for converting the precursors to active GA isoforms were up-regulated, while the transcript level of the enzyme gibberellins 2-beta-dioxygenese 8 (GA2ox8) (EC1.14.11.13) for inactivation of GAs was decreased in FER (Fig.10a). These results suggest that a higher accumulation of active GAs in FER than in ER. We subsequently determined 10 types of GA molecules, including GA 1, 3, 4, 7, 9, 15, 19, 20, 24 and 53, in TB, ER and FER samples by LC-MS-MS. All the GAs but GA4 were detected in ramie samples. The active form GA7 had a very low concentration, while GA1 was the most abundant active GA. GA9 was the most abundant precursor. Our results showed that FER samples had the most abundant GA precursors and active GA molecules, while the TB samples had lowest contents of GAs (Fig.10b).

## Discussions

### Secondary cell wall biosynthesis is enhanced in the stem bark in ramie

In the Acid Growth Theory, auxin plays a critical role in triggering and the formation of an acidic cell wall environment [29]. Plant morphogenesis involves cell wall biosynthesis and the enlargement of cell wall, which requires cell wall loosening by the proteins or enzymes such as expansins, xyloglucan endotransglycosylases, and GBALs, the deposition of cell wall materials such as cellulose, and the modifications of the cell wall components [21, 29]. Although GO and KEGG did not identify auxin signaling pathways in the DEGs, 29 up-regulated and 7 down-regulated unigenes in FER comparing to ER were found to be auxin related genes, including calcium-binding protein PBP1, auxin-responsive protein SAUR22/36, IAA18, WAT1-related proteins, MYB44, putative indo-3-acetic acid-amido synthetase GH3.9, etc. (Additional file 1: Table S6). Another transcription factor Myb 26, functioning in crosstalk of auxin and jasmonic acid, responding to GA and playing a role in fine-tuned regulation of fiber or secondary cell wall synthesis [16, 30, 31], exhibited a higher expression in ER than in FER.

In addition to auxin related genes, we also identified proteins and enzymes important for cell wall construction among the DEGs. A number of unigenes involved in secondary cell wall synthesis are up-regulated in both ER and FER (Fig. 4, Additional file 1: Table S2). The CesA 3 and 8 are among the cell wall related DEGs (Additional file 1: Table S2). There are 9 CesA genes orthologous to Arabidopsis identified in ramie from our RNA-seq analysis, but only CesA 3 and CesA 8 show up-regulation in both ER and FER, which indicates that these 2 genes might be involved in ramie’s secondary cell wall biogenesis during fiber development. Coincidently, CesA 8 from Arabidopsis was found to play a role in secondary cell wall synthesis in flax [1]. In addition to cellulose synthases, FLAs and BGALs have also been reported to be involved in the formation of cellulose rich gelatinous fibers [1, 16–18]. Among the DEGs in the cluster 2, FLA 11/12 and BGAL 3/9 are up-regulated in ER and FER (Additional file 1: Table S2). In addition, PMEs/PMEIs and the enzymes for the synthesis of cell wall components are also among the DEGs in the cluster 2 (Additional file 1: Table S2). These enzymes or protein factors were reported to play distinct roles in modulation or biosynthesis of secondary cell wall [7–9, 32, 33].

The analysis between ER and FER demonstrated that, although 20 secondary cell wall-related unigenes are down-regulated in FER (Fig. 6b), as many as 84 unigenes for secondary cell wall synthesis are up-regulated in FER when compared with ER (Additional file 1: Table S7). These results indicate that, although secondary cell wall biogenesis is strengthened in both ER and FER, FER exhibits differences from ER in the expression of genes for secondary cell wall biosynthesis, and more enzymes might be required in FER for the thickening and enlarge of the fiber cell wall (Fig. 1d). In addition to CesA 3/8, FLA11/12, BGAL 3/9, PME 13/28/35 and several other factors that are up-regulated in both ER and FER, the genes for other PMEs, BGALs, an FLA 8, subtilisin-like proteases (SBTs), some leucine-rich repeat extension-like proteins (LRXs), peroxidase 47/64, laccase-4 (IRX12), pathogenesis-related protein 5 etc. show higher expression level in FER than in ER. FLAs contain a cell adhesion fasciclin (FAS) domain. Expression of some FLAs has been shown to be correlated with the onset of secondary wall and cellulose synthesis in Arabidopsis stem, and with tension wood formation in the stem and branch in *Populus tremula* (L.) [21, 34] Mutations in FLA genes result in altered stem biomechanics with reduced tensile strength and elasticity, as well as altered cell wall architecture and composition [20]. While pectin is another important component in ramie’s fiber cell wall, PMEs act via demethylesterification of cell wall pectin, which was identified to function in the flax phloem fiber development [32]. PMEs/PMEIs were also shown to carry out endohydrolysis of the N-glycosidic bond at one specific adenosine on the 28S rRNA and inactivate the ribosome [21], which may account for the decreased expression of translation factors in FER (Fig. 6b, Additional file 2: Figure S3B).

### Phloem development related genes are upregulated in the FER region

Both the morphological observation and GO analysis of DEGs comparing ER with FER indicate that secondary phloem formation is related to the maturation of stem bark. We found that the contigs of five protein encoding genes involving 13 assembled unigenes are possibly responsible for enhanced secondary phloem development in FER. These proteins include sieve element occlusion A (SEOA), SEOB, Myb family transcription factor Altered Phloem Development (APL), protein DA1-related 2 (DAR 2) and UPF0503 (Table 1). SEOA and SEOB are two phloem filament proteins, and their orthologues in Arabidopsis are required for the formation of phloem filaments. Phloem filaments could only be detected when both SEO proteins are present in Arabidopsis [35–37]. APL transcription factor is required for phloem identity, and has a dual role in promoting phloem differentiation and repressing xylem differentiation during vascular development [36–38]. The downstream targets of APL include NAC45 and NAC86, and these two proteins are involved in enucleation, organelles reorganization and cytosol degradation during sieve element cell differentiation [37, 39]. Consistently, NAC86 is also significantly up-regulated in the FER with a change of more than 4-fold. DAR 2 is involved in root phloem development and is an essential component for early phloem development and long-distance transport of phloem contents in Arabidopsis [36, 40]. The unigene CL252Contig2 is likely to encode the protein UPF0503, whose orthologue in Arabidopsis is the polarly localized membrane-associated protein OCTOPUS (OPS), which is initially expressed in provascular cells, and upon vascular cell type specification, it becomes restricted to the phloem cell lineage. The *ops* mutants display a reduction in the complexity of the cotyledon vascular pattern and exhibit discontinuous phloem differentiation, whereas OPS overexpressors show accelerated progress of cotyledon vascular patterning and phloem differentiation [36, 37, 41]. The expression of some DEGs related to phloem or vascular development, including APL and DAR2, which termed as Phloem 1 and Phloem 2, was further verified by RT-qPCR. The results showed significantly increases in the expression of these genes in FER (Additional file 2: Figure S3A and Additional file 1: Table S8).

**Table 1:** Up-regulated unigenes (FER vs. ER) in phloem and vasculature development

Unigene	Base Mean Exp.		log2	SWISSPROT Description
	ER	FER	F C	
Phloem development				
CL18144Contig1	9.5	44.8	2.2	Protein SIEVE ELEMENT OCCLUSION A
CL23122Contig1	35.1	175.4	2.3	Protein SIEVE ELEMENT OCCLUSION A
CL26574Contig1	6.2	28.5	2.2	Protein SIEVE ELEMENT OCCLUSION A
CL37212Contig1	67.9	347.7	2.4	Protein SIEVE ELEMENT OCCLUSION A
CL13271Contig1	33.8	205.0	2.6	Protein SIEVE ELEMENT OCCLUSION B
CL18310Contig1	8.8	34.1	2.0	Protein SIEVE ELEMENT OCCLUSION B
CL2296Contig1	50.2	158.0	1.7	Protein SIEVE ELEMENT OCCLUSION B
CL19357Contig1	36.9	129.4	1.8	Protein SIEVE ELEMENT OCCLUSION B
comp53409_c0_seq1_2	60.7	282.6	2.2	Protein SIEVE ELEMENT OCCLUSION B
CL1969Contig2	70.9	167.7	1.2	Myb family transcription factor APL
CL216Contig3	424.0	953.7	1.2	Myb family transcription factor APL
CL28929Contig1	26.9	94.2	1.8	Protein DA1-related 2
CL40627Contig1	60.6	171.5	1.5	Protein DA1-related 2
CL252Contig2	23.1	94.5	2.0	UPF0503 protein At3g09070, chloroplastic
Vasculature development				
CL12072Contig2	3.6	22.2	2.6	Ethylene-responsive transcription factor 2 ERF2
CL153Contig1	1105.8	3191.3	1.5	Ethylene-responsive transcription factor ERF109
CL16199Contig1	14.1	56.9	2.0	WUSCHEL-related homeobox 4 WOX4
CL1822Contig1	76.3	371.1	2.3	Ethylene-responsive transcription factor 1A ERF1A

### Ethylene might be involved in vascular maturation in ramie’s stem bark

We also identified 4 unigenes including WUSCHEL-related homeobox 4 (WOX4) and other three ethylene-responsive transcription factors (ERFs) (Table 1) that are involved in vasculature development. WOX4 functions downstream of the dodeca-peptide TE differentiation inhibition (TDIF) factors and its receptor PHLOEM INTERCALATED WITH XYLEM (PXY), and promotes cambial cell proliferation [42–44]. The mutation of *WOX4* represses procambium proliferation in the hypocotyl of 7-day-old seedlings in *Arabidopsis* [42–44]. The significant up-regulation of *ERF109*, *WOX4* and *ERF1A* in FER was also verified by RT-qPCR, which named as Vascul1, Vascul2 and Vascul3, respectively (Additional file 2: Fig. S3A and Additional file 1: Table S8). The up-regulated ethylene activating pathway in FER might be a parallel event to WOX4-PXY signal for phloem or vascular development. PXY is a receptor kinase and functions in ordered and coordinated cell divisions in the procambium [44, 45]. Twelve members of AP2/ERF family, including *ERF109*, *ERF11*, *ERF104*, *ERF018*, *ERF1*, *ERF2*, *ERF5* and *ERF6*, were identified to be up-regulated in *pxy* mutant, which was thought to compensate the loss of function of *PXY* gene in Arabidopsis. Especially, loss of function mutations of both *ERF 109* and *ERF 018*, together with the *pxy* mutation, significantly reduced vascular cell numbers [45]. ERF1 was also found to be a key factor for vascular cell division responding to ethylene signal [45]. Additionally, the base of stems of Arabidopsis exhibits larger fold changes in the gene expression of *ERF109*, *ERF11*, *AtERF1* and *ERF018* than that observed in the middle of stems [45]. Since the FER is closer to the base position of the whole stem of ramie, it is likely that the genes activating ethylene pathway are required for the mechanical and physiological properties of this region. Moreover, ethylene is induced during tension wood formation in poplar, and several factors in the ethylene signaling pathway were identified to be up-regulated during tension wood formation [46]. Therefore, the activation of ethylene pathway or ERFs expression could be an important molecular basis for ramie’s fiber differentiation and development. The up-regulation of ethylene pathway and increased *WOX4* expression could partially explain the burst of secondary phloem formation or cambia activity in the relatively mature part of ramie stem.

Furthermore, ethylene might play a role in fiber cell elongation. Ethylene signaling pathway has been implicated in fiber elongation in cotton [47, 48]. Exogenously applied ethylene promotes robust fiber cell expansion, while the ethylene biosynthetic inhibitor L-(2-aminoethoxyvinyl)-glycine (AVG) specifically suppresses fiber growth in cotton [47]. The expression of ethylene biosynthesis enzyme 1-aminocyclopropane-1-carboxylic acid oxidase (ACO) genes is significantly up-regulated during cotton fiber development [47]. The cotton 1-aminocyclopropane-1-carboxylic acid synthase 2 (ACS2) is phosphorylated by Ca^2+^-dependent protein kinase 1 (CPK1), which increased the ACS activity, leading to elevated ethylene biosynthesis during rapid fiber elongation [48]. Although we did not detect up-regulation of ACOs in both ER and FER, activation of the genes in ethylene pathway identified in our analysis suggests that ethylene may also contribute to fiber elongation in ramie.

### The expression of GA synthesis enzymes and GA contents altered between ER and FER

GA is another hormone playing a role in cellulose-rich fiber development in plant. A recent study of transcriptomic profiling of hemp bast fibres at different developmental stages revealed that GA biosynthesis is significantly increased in the bark of bottom stem of hemp [2]. In addition, recent studies in ramie have also supported the role of GA in bast fiber development [4, 49], and overexpression of the Arabidopsis gibberellic acid 20 oxidase (AtGA20ox) enhances vegetative growth and fiber quality in kenaf (*Hibiscus cannabinus* L.) plants [50, 51]. GA was also proposed to function through promoting cambia cell proliferation to increase the cell amount of phloem tissue in Arabidopsis [52]. Our KEGG analysis and endogenous GA detection indicated a difference in GA biosynthesis and accumulation between ER and FER. GA1 is the most abundant and could be the major active form of GAs to contribute to phloem development in ramie. All the detected GAs showed increasing concentrations along the stem from the top to the base, which suggests that GA might play an important role in the maturation of fibers.

## Conclusions

**Fig. 11:**
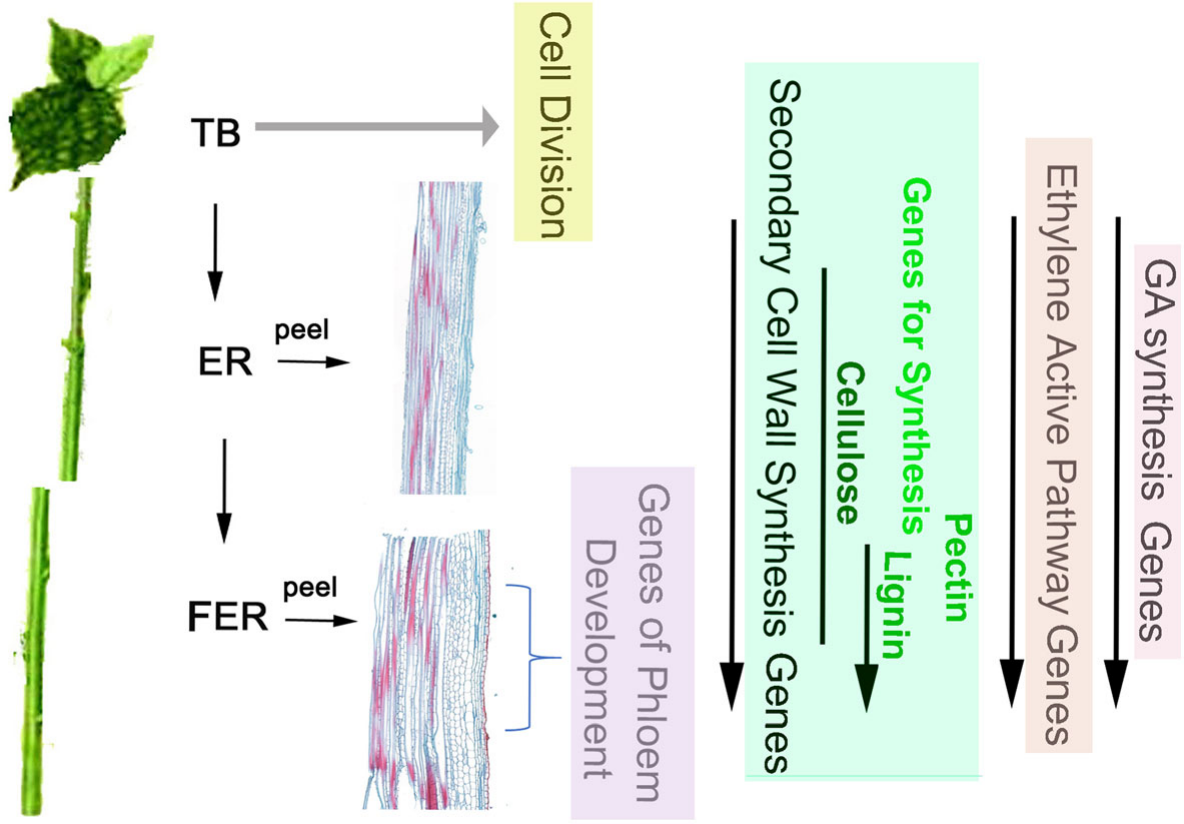
A schematic showing gene expression in TB, ER and FER in Ramie. Gene activation in FER for phloem development is clearly evidenced in our study. Secondary cell wall synthesis genes are up-regulated in both ER and FER but more activated in FER. The genes in the ethylene pathway and gibberellins acids biosynthesis are activated during the maturation of ramie stem

In summary, our findings revealed a burst of phloem formation and secondary cell wall synthesis in the fully elongated regions of the stem with relatively mature fibers in the stem bark of ramie. We found that two types of phytohormones, ethylene and gibberellin, might be involved in the development of ramie’s phloem fiber tissues (Fig. 11). However, more studies are needed to elucidate the molecular basis of secondary phloem development. Nonetheless, our study could provide valuable genomic data and gene expression profiles related to the fiber formation in ramie for improvement of this important fiber crop.

## Methods

### Materials

ZhongZhu No. 1 ramie variety was planted in the green house, originated from cutting propagation of plant in farm of Institute of Ramie, Hunan Agricultural University, Changsha, China. The plant was identified by Ramie’s Germplasm Resource Description Standards GB/T 2659 of Crop Germplasm Resource Infrastructure in China (www.cgris.net). And Yucheng Jie was responsible for identification of the ramie variety ZhongZhu No.1. Ramie’s shoot of the third season growth including CAM and its connected stem with green surface was harvested. The shoot was then truncated into 3 sections: top bud (TB), internodes elongating region (ER) of stem and internodes fully elongated region (FER) of stem. The elongating or fully elongated regions were judged by the length between adjacent internodes. When the length between the internodes was 2 cm longer than the upper ones but 2 cm shorter than the lower ones, this section was considered as the elongating region. Below the elongating region was considered as fully elongated region. All the leaves and flowers associated with the shoot were removed, and the bark was peeled from these two stem fragments. The top bud and two bark fragments, which termed as TB, ER and FER were used for further experiments (Fig. 1a).

### Tissue sections and images collection

Tissue materials of TB, ER and FER were fixed in FAA (formaldehyde, acetic acid, 70% ethanol) until further processing. The fixed materials were then embedded by paraffin. Microscope slides with transverse or longitudinal sections were prepared using a rotation microtome and following standard protocols. The sections were stained with safranin-fast-green or aniline blue, and the stained sections were then scanned by Pannoramic 250/MIDI (Wuhan servicebio technology company). Images were collected by caseviewer software provided by Wuhan servicebio technology company.

### RNA extraction and RNA sequencing

Total RNA was extracted from TB, ER and FER using TRIzol reagent kit (Invitrogen, Carlsbad, CA, US) following the manufacturer’s protocol. The mRNAs were enriched by magnetic beads coated with Oligo (dT), and then randomly fragmented by ultrasound. The platform of Illumina HiSeq 2500 was adopted to carry out RNA sequencing (RNA-seq) by OE biotech company (Shanghai). The sequencing data have been deposited in the NCBI Sequence Read Archive (SRA, https://www.ncbi.nlm.nih.gov/sra) with accession number SRP 199269.

### RNA-seq data analysis

De novo transcriptome was assembled in OE biotech company (Shanghai). Briefly, the total reads were assembled into transcripts using paired-end method of the Trinity software (trinityrnaseq_r20131110) [53], and the unigenes were identified by selecting the longest transcripts among the overlapping sequences. The TIGR Gene Indices clustering tools (TGICL) were then used to remove the redundant transcripts [54]. The de novo assembled transcriptome was then used as the reference for the subsequent analyses. Unigenes were annotated by searching the databases of NR, SWISSPROT and KOG using Basic Local Alignment Search Tool (BLAST) [55]. The expression of unigenes was calculated and subsequently normalized to RPKM [56]. The uniform screening conditions for DEGs were *p* < =0.05, and fold change >2 or fold change <0.5. Then the DEGs were submitted to perform GO and KEGG analyses [57]. VENN diagram was made using a website tool (http://bioinformatics.psb.ugent.be/webtools/Venn/). And the K-means clustering algorithm was adopted to analyze the expression pattern.

### RT-qPCR

Total RNA was extracted from TB, ER and FER using TRIzol reagent kit (Invitrogen, Carlsbad, CA, US) following the manufacturer’s protocol. After TURBO DNase I (Ambion) treatment, 2 μg of RNA was subjected to reverse transcription reaction using the TransScript One-Step gDNA Removal and cDNA Synthesis SuperMix kit (TransGen Biotech). Then cDNAs were used as templates for qPCR with Green qPCR SuperMix (TransGen Biotech) in a CFX96 real-time PCR detection system (Bio-RAD). Ubiquitin-conjugating enzyme E2 (CL1514Contig1) was chosen as a reference according to our RNA-seq data. All of the primers are listed in Additional file 1: Table S8. All the reactions were done in triplicates.

### Detection of GAs by LC-ESI-MS/MS

Fresh plant materials of TB, ER and FER were harvested and quick-frozen in liquid nitrogen. 200 mg of powdered sample was used for extraction of GAs by using 1500 ul 70% (v/v) acetonitrile overnight at 4 °C. The supernatants were passed through the SPE cartridge (300 mg, 6 ml, Agela) and evaporated to dryness under nitrogen gas stream. Then the samples were reconstituted in 200 ul 80% (v/v) methanol and filtrated through 0.22 um pore size (Anpel). The sample extracts were analyzed using an LC-ESI-MS/MS system (HPLC, Shim-pack UFLC SHIMADZU CBM30A system, MS, Applied Biosystems 6500 Triple Quadrupole). The analytical conditions were as follows, HPLC: column, Waters ACQUITY UPLC HSS T3 C18 (1.8 μm, 2.1 mm*100 mm); solvent system, water (0.04% acetic acid): acetonitrile (0.04% acetic acid); gradient program, 80:5 V/V at 0 min, 50:95 V/V at 10.0 min, 35:95 V/V at 11.0 min, 25:5 V/V at 11.1 min, 10:5 V/V at 14.0 min; flow rate, 0.35 mL/min; temperature, 45 °C; injection volume: 5 μL. The effluent was alternatively connected to an ESI-triple quadrupole-linear ion trap (Q TRAP)-MS. API 6500 Q TRAP LC/MS/MS System, equipped with an ESI Turbo Ion-Spray interface, operating in a negative ion mode and controlled by Analyst 1.6.3 software (AB Sciex). The ESI source operation parameters were as follows: ion source, turbo spray; source temperature 550 °C; ion spray voltage (IS)- 4500 V; curtain gas (CUR) were set at 35.0 psi; the collision gas (CAD) was medium. DP and CE for individual MRM transitions was done with further DP and CE optimization. A specific set of MRM transitions were monitored for each period according to the plant hormones eluted within this period. All of the gibberellin standards were purchased from Olchemim Ltd. (Olomouc, Czech Republic) and Sigma (St. Louis, MO, USA).

## Supplementary information

**Additional file 1: Table S1.** FER had thicker fiber cell wall than ER. **Table S2.** Up-regulated DEGs in both ER and FER vs. TB involving in cell wall synthesis. **Table S3.** The most of up-regulated transcription factors in FER were ethylene responsive. **Table S4.** Ethylene active pathway up-regulated in FER comparing with ER. **Table S5.** KEGG enrichment top 20. **Table S6.** DEGs between ER and FER relative to Auxin. **Table S7.** DEGs between ER and FER involving in cell wall synthesis. **Table S8.** RT-qPCR verified unigenes.

**Additional file 2: Figure S1.** GO analysis of the DEGs between TB and ER. **Figure S2.** GO analysis of the DEGs between TB and FER. **Figure S3.** RT-qPCR detection of the selected DEGs among TB, ER and FER.

## Data Availability

The sequencing data are deposited in NCBI Sequence Read Archive (SRA, http://www.ncbi.nlm.nih.gov/Traces/sra) with accession number of SRP199269.

## Abbreviations

ACO: 1-aminocyclopropane-1-carboxylic acid oxidase
ACS2: 1-aminocyclopropane-1-carboxylic acid synthase 2
AtGA20ox: *Arabidopsis thaliana* gibberellic acid 20 oxidase
BGAL: Beta-galactosidase
BLAST: Basic Local Alignment Search Tool
CesA: Cellulose Synthase A
CPK1: Ca2 + −dependent protein kinase 1 (CPK1)
DEG: Differentially expressed genes
ER: Internode Elongating Region
ERFs: Ethylene respond factors
FER: Internode fully elongated region
FLA: Fasciclin-like Arabinogalactan Protein
GAs: Gibberellin acids
GO: Gene Ontology
KEGG: Kyoto Encyclopedia of Genes and Genomes
NGS: Next generation sequencing
PMEs/PMEIs: Pectinesterase/pectinesterase inhibitors
RNA-seq: RNA sequencing
SAM: Shoot apical meristem
SBTs: Subtilisin-like proteases
TB: Top Bud
