# Supplementary material for: Comparative transcriptomics of stem bark reveals genes associated with bast fiber development in Boehmeria nivea L. gaud (ramie)
Source: BMC Genomics. 2020 Jan 13;21:40. doi: 10.1186/s12864-020-6457-8 (PMC6958601; doi:10.1186/s12864-020-6457-8)
Supplement: Supplementary file 1 — Additional file 1: Table S1. FER had thicker fiber cell wall than ER. Table S2. Up-regulated DEGs in both ER and FER vs. TB involving in cell wall synthesis. Table S3. The most of up-regulated transcription factors in FER were ethylene responsive. Table S4. Ethylene active pathway up-regulated in FER comparing with ER. Table S5. KEGG enrichment top 20. Table S6. DEGs between ER and FER relative to Auxin. Table S7. DEGs between ER and FER involving in cell wall synthesis. Table S8. RT-qPCR verified unigenes. [file 12864_2020_6457_MOESM1_ESM.pdf]

Supplemental table 1

FER had thicker fiber cell wall than ER

	Lumen Diameter (μm)	T-test	Cell Wall Thickness (μm)	T-test	Cell Diameter (μm)	T-test
FER	22.74 ±6.44	0.02	5.38 ±1.22	<0.01	36.58 ±7.09	0.16
ER	26.56 ±6.33		1.87 ±0.31		34.61 ±6.46	

Supplemental table 2

Up-regulated DEGs in both ER and FER *vs*. TB involving in cell wall synthesis

Unigene	Base Mean Expression			SWISSPROT Description
	TB	ER	FER	
CL32Contig14	363	2116	1166	Cellulose synthase A catalytic subunit 8
CL32Contig2	148	1669	1140	Cellulose synthase A catalytic subunit 8
comp47834_c6_seq2_5	400	2866	1511	Cellulose synthase A catalytic subunit 3
CL1318Contig1	204	4668	3727	Fasciclin-like arabinogalactan protein 12
CL16940Contig1	108	1007	883	Fasciclin-like arabinogalactan protein 12
CL19276Contig1	12	108	79	Fasciclin-like arabinogalactan protein 12
CL23Contig8	587	12083	9612	Fasciclin-like arabinogalactan protein 12
CL25112Contig1	117	1018	1032	Fasciclin-like arabinogalactan protein 12
CL5588Contig1	238	5802	4777	Fasciclin-like arabinogalactan protein 11
CL2132Contig2	18	191	111	Fasciclin-like arabinogalactan protein 11
CL23Contig9	28	341	446	Fasciclin-like arabinogalactan protein 11
CL36Contig2	28	276	116	Transcription factor MYB26
CL2547Contig1	2664	10026	6610	Probable glucuronoxylan glucuronosyltransferase IRX7
CL1242Contig2	43	220	265	Protein trichome birefringence-like 19
CL1Contig120	245	736	617	Protein trichome birefringence-like 19
CL12537Contig1	1615	7439	5971	Protein EXORDIUM-like 2
CL13538Contig1	142	1242	677	Probable galacturonosyltransferase-like 4
comp51775_c0_seq6_4	562	1388	1561	Probable galacturonosyltransferase 15
CL14235Contig1	14	98	64	UDP-arabinopyranose mutase 3
CL1525Contig3	1746	5015	4588	SNF1-related protein kinase regulatory subunit gamma-1
CL1525Contig5	357	1198	1068	SNF1-related protein kinase regulatory subunit gamma-1
CL1695Contig1	1457	5273	4350	Glucan endo-1,3-beta-glucosidase 14
CL19522Contig1	108	397	365	Pectinesterase/pectinesterase inhibitor
CL7925Contig2	110	314	325	Pectinesterase/pectinesterase inhibitor
CL208Contig3	315	1455	903	Probable pectinesterase/pectinesterase inhibitor 13
CL673Contig1	3214	16884	15725	Probable pectinesterase/pectinesterase inhibitor 35
CL749Contig3	128	715	499	Putative pectinesterase/pectinesterase inhibitor 28
CL1Contig162	12	60	64	Protein trichome birefringence-like 37
CL31344Contig1	102	208	269	Protein trichome birefringence-like 19
CL218Contig1	298	748	607	ETHYLENE INSENSITIVE 3-like 1 protein
CL22Contig13	628	1321	1658	Probable galacturonosyltransferase 15
comp38070_c0_seq7_3	195	751	1037	Probable galacturonosyltransferase 15
CL24576Contig1	114	283	269	Peroxidase 24
CL2662Contig2	7	42	36	Pathogenesis-related protein 5
CL284Contig1	138	536	376	Acidic endochitinase
CL284Contig4	6	33	28	Acidic endochitinase
CL31245Contig1	730	4108	1491	Snakin-2
CL3337Contig1	7	183	141	Mulatexin
CL34316Contig1	127	344	303	Putative callose synthase 8
CL34Contig7	230	547	772	Probable xyloglucan glycosyltransferase 12

CL4236Contig1	55	156	143	Polygalacturonase At1g48100
CL4658Contig1	12	252	488	Beta-fructofuranosidase, insoluble isoenzyme 1
CL5888Contig1	516	1455	1098	Subtilisin-like protease SBT5.3
comp48569_c0_seq4_4	0	24	11	Subtilisin-like protease SBT5.3
CL6571Contig1	3150	9700	6861	Callose synthase 2
CL85Contig9	463	1245	959	Alpha-L-arabinofuranosidase 1
CL969Contig2	144	1576	533	Glucomannan 4-beta-mannosyltransferase 9
comp51626_c1_seq12_4	349	2676	1347	Glucomannan 4-beta-mannosyltransferase 9
comp35113_c0_seq1_2	22	138	71	D-alanine--D-alanine ligase
comp47433_c0_seq177_4	0	25	14	D-alanine--D-alanine ligase
comp42054_c0_seq2_3	4354	11922	15184	Probable xyloglucan endotransglucosylase/XTH23
comp47435_c0_seq1_1	2011	6168	8102	Probable xyloglucan endotransglucosylase/XTH23
comp45402_c0_seq2_4	151	516	452	Glucan endo-1,3-beta-glucosidase 5
comp48380_c1_seq1_1	59	724	270	Glucomannan 4-beta-mannosyltransferase 9
comp50396_c0_seq1_4	1385	20465	12529	Beta-galactosidase 3
comp51476_c1_seq55_5	574	1629	1290	Beta-galactosidase 9

Supplemental table 3

The most of up-regulated transcription factors in FER were ethylene responsive

Unigene	Base Mean Expression			SWISSPROT Description
	TB	ER	FER	
**CL18694Contig1**	38	21	98	Ethylene-responsive transcription factor 1
**CL6145Contig1**	54	22	140	Ethylene-responsive transcription factor 1
**CL12072Contig1**	109	49	253	Ethylene-responsive transcription factor 2
**CL58Contig3**	59	69	144	Ethylene-responsive transcription factor 3
**comp30422_c1_seq1_2**	724	3484	8107	Ethylene-responsive transcription factor 5
**comp48253_c1_seq1_2**	480	2851	7339	Ethylene-responsive transcription factor 5
**CL8886Contig1**	167	398	1447	Ethylene-responsive transcription factor ERF017
**CL15626Contig1**	283	387	868	Ethylene-responsive transcription factor ERF061
**CL8568Contig1**	81	58	236	Ethylene-responsive transcription factor ERF071
**CL8568Contig2**	63	69	229	Ethylene-responsive transcription factor ERF071
**CL153Contig1**	705	1106	3180	Ethylene-responsive transcription factor ERF109
**CL4057Contig1**	14	23	106	Ethylene-responsive transcription factor ERF022
**comp34354_c0_seq1_6**	12	14	62	Ethylene-responsive transcription factor ERF022
**CL4Contig44**	731	1227	2447	Ethylene-responsive transcription factor ERF053
**CL38180Contig1**	136	423	1111	Ethylene-responsive transcription factor RAP2-4
**CL29932Contig1**	1746	3448	6914	Ethylene-responsive transcription factor RAP2-13
**comp34163_c1_seq1_3**	1760	2676	5411	Transcription factor MYB44
CL1316Contig3	99	153	371	NAC domain-containing protein 2
CL2879Contig3	428	450	1075	NAC domain-containing protein 2
comp42223_c0_seq3_2	164	316	667	NAC domain-containing protein 13
CL29473Contig1	20	49	138	NAC domain-containing protein 90
CL16564Contig1	20	16	105	Transcription factor bHLH35
comp46971_c0_seq1_4	121	195	433	Transcription factor bHLH62
CL4875Contig1	111	142	590	Transcription factor bHLH113
CL18620Contig1	1509	2182	5120	Transcription factor MYC2
CL216Contig3	463	424	949	Myb family transcription factor APL
comp44992_c0_seq2_6	131	155	564	Probable WRKY transcription factor 30
CL26613Contig1	27	50	155	Probable WRKY transcription factor 33
CL3295Contig1	10	12	61	Probable WRKY transcription factor 33
CL39283Contig1	429	991	3447	Probable WRKY transcription factor 40
CL929Contig4	173	634	1907	Probable WRKY transcription factor 41
CL5708Contig2	144	200	698	Probable WRKY transcription factor 70
CL325Contig3	86	59	173	Zinc finger protein 7
CL343Contig2	30	40	97	B-box zinc finger protein 32
CL404Contig1	0	5	31	Transcription factor UNE10
CL4989Contig1	345	269	693	Transcription factor PCL1
CL530Contig2	29	21	73	SNF1-related protein kinase regulatory subunit beta-1
CL8675Contig1	142	217	454	Agamous-like MADS-box protein AGL12
comp48092_c2_seq1_1	23	117	314	Zinc finger protein ZAT10

Supplemental table 4

Ethylene active pathway up-regulated in FER comparing with ER

Unigene	Base Mean Exp.		log2	SWISSPROT Description
	ER	FER	FC	
CL18694Contig1	21.45	97.93	2.19	Ethylene-responsive transcription factor 1 ERF1
CL6145Contig1	21.77	141.06	2.70	Ethylene-responsive transcription factor 1 ERF1
CL1822Contig1	76.25	371.15	2.28	Ethylene-responsive transcription factor 1A ERF1A
CL24411Contig1	2.64	21.17	3.00	Ethylene-responsive transcription factor 1B ERF1B
CL12072Contig1	49.40	254.11	2.36	Ethylene-responsive transcription factor 2 ERF2
CL12072Contig2	3.61	22.20	2.62	Ethylene-responsive transcription factor 2 ERF2
CL58Contig3	69.00	144.95	1.07	Ethylene-responsive transcription factor 3 ERF3
CL4811Contig1	23.19	58.71	1.34	Ethylene-responsive transcription factor ERF003
CL9Contig17	3129.93	8791.78	1.49	Ethylene-responsive transcription factor 5 ERF5
comp30422_c1_seq1_2	3483.73	8135.80	1.22	Ethylene-responsive transcription factor 5 ERF5
comp48253_c1_seq1_2	2850.84	7357.97	1.37	Ethylene-responsive transcription factor 5 ERF5
CL8886Contig1	398.06	1450.38	1.87	Ethylene-responsive transcription factor ERF017
CL4057Contig1	22.84	107.14	2.23	Ethylene-responsive transcription factor ERF022
comp34354_c0_seq1_6	14.11	62.87	2.16	Ethylene-responsive transcription factor ERF022
CL4Contig44	1226.62	2455.37	1.00	Ethylene-responsive transcription factor ERF053
CL15626Contig1	387.14	871.12	1.17	Ethylene-responsive transcription factor ERF061
CL8568Contig1	58.48	237.07	2.02	Ethylene-responsive transcription factor ERF071
CL8568Contig2	68.95	230.41	1.74	Ethylene-responsive transcription factor ERF071
CL153Contig1	1105.78	3191.26	1.53	Ethylene-responsive transcription factor ERF109
CL29932Contig1	3448.19	6938.54	1.01	Ethylene-responsive transcription factor RAP2-13
CL38180Contig1	422.65	1114.29	1.40	Ethylene-responsive transcription factor RAP2-4
comp39410_c0_seq1_2	1353.14	2719.36	1.01	Mitogen-activated protein kinase kinase 9 MKK9
CL54Contig2	480.58	1038.39	1.11	Dehydration-responsive element-binding protein 3 DREB3
CL14901Contig1	54.29	125.57	1.21	Molybdenum cofactor sulfurase MCSU3
CL55Contig10	73.47	158.89	1.11	Lipase-like PAD4

Supplement Table 5

KEGG enrichment top 20

KEGG ID	term	P-value	Enrichment_score
ko00500	Starch and sucrose metabolism	2.50E-05	2.428748479
ko00020	Citrate cycle (TCA cycle)	5.45E-05	3.021578009
ko00910	Nitrogen metabolism	0.000647	3.28057041
ko00270	Cysteine and methionine metabolism	0.000656	2.275540169
ko00630	Glyoxylate and dicarboxylate metabolism	0.000919	2.423148598
ko03030	DNA replication	0.00117	3.02821884
ko04110	Cell cycle	0.001563	2.105178873
ko02020	Two-component system	0.001598	3.844418449
ko00710	Carbon fixation in photosynthetic organisms	0.001865	2.252504331
ko00620	Pyruvate metabolism	0.001897	2.180125905
ko00591	Linoleic acid metabolism	0.001994	3.021578009
ko04111	Cell cycle - yeast	0.003304	2.118249106
ko03010	Ribosome	0.004294	1.565282104
ko01200	Carbon metabolism	0.0045	1.572644578
ko00940	Phenylpropanoid biosynthesis	0.00477	1.763747532
ko04113	Meiosis - yeast	0.005101	2.14799253
ko00904	Diterpenoid biosynthesis	0.005904	2.733808675
ko00040	Pentose and glucuronate interconversions	0.007992	1.968342246
ko00945	Stilbenoid, diarylheptanoid and gingerol biosynthesis	0.010685	2.016744104
ko00350	Tyrosine metabolism	0.013824	2.013077297

Supplemental table 6

DEGs between ER and FER relative to Auxin

Unigene ID	log2FoldChange	P-val	SWISS Description
CL109Contig1	2.853767	0.029841	Calcium-binding protein PBP1
CL39522Contig1	3.491533	0.000219	Calcium-binding protein PBP1
CL2495Contig1	1.687678	0.001088	Calcium-binding protein PBP1
CL11140Contig1	2.428512	0.001101	Auxin-responsive protein SAUR36
CL19829Contig1	2.532322	0.016734	Auxin-responsive protein SAUR22
CL3166Contig1	1.597525	0.045933	Auxin-responsive protein IAA18
CL14901Contig1	1.209904	0.01157	Molybdenum cofactor sulfurase
CL1592Contig2	1.763622	0.000165	VAN3-binding protein
CL16880Contig1	1.492669	0.003771	Putative auxin efflux carrier component 5
CL5307Contig1	2.468217	0.011837	Putative auxin efflux carrier component 8
CL1Contig144	1.329512	0.000525	Putative indole-3-acetic acid-amido synthetase GH3.9
CL1Contig93	1.178152	0.008761	Putative indole-3-acetic acid-amido synthetase GH3.9
comp49368_c0_seq1_4	1.287212	5.42E-05	Putative indole-3-acetic acid-amido synthetase GH3.9
comp49508_c1_seq2_2	1.536928	0.001478	Putative indole-3-acetic acid-amido synthetase GH3.9
CL21136Contig1	2.209705	0.001046	Transcription factor MYB44
CL22382Contig1	1.354032	0.021886	WAT1-related protein At3g30340
CL23613Contig1	2.590587	0.005661	WAT1-related protein At2g39510
CL24156Contig1	1.665487	6.60E-05	WAT1-related protein At3g30340
comp47833_c0_seq2_4	1.560987	0.000661	WAT1-related protein At3g30340
CL23308Contig1	1.493258	0.046919	Probable glutathione S-transferase parA
CL24238Contig1	1.190705	0.025018	ABC transporter B family member 11
CL2703Contig1	2.357473	1.71E-05	Protein TORNADO 2
CL3965Contig1	1.439976	0.022875	Subtilisin-like protease SBT5.3
CL6Contig42	1.082299	0.046019	UDP-glycosyltransferase 74D1
CL7287Contig2	1.380356	0.024293	Auxin transporter-like protein 2
comp48184_c0_seq1_4	1.228378	0.002779	Auxin transporter-like protein 2
comp34163_c1_seq1_3	1.021186	0.011756	Transcription factor MYB44
comp50528_c1_seq1_5	1.320521	0.005015	Pyrophosphate-energized vacuolar membrane proton pump 1
comp53814_c1_seq1_2	1.096749	0.00041	Flavonoid 3'-monooxygenase
CL10280Contig1	-1.56233	0.045929	Auxin-induced protein X10A
CL1608Contig1	-2.7175	0.00244	NAC domain-containing protein 21/22
CL16733Contig2	-2.92396	0.021279	Serine/threonine-protein kinase D6PKL2
CL20Contig18	-1.25689	7.48E-05	Protein AUXIN SIGNALING F-BOX 2
CL24033Contig1	-4.56125	0.000619	Probable indole-3-pyruvate monooxygenase YUCCA4
CL6748Contig1	-1.49502	0.000665	WAT1-related protein At1g70260
CL6Contig28	-1.37347	0.030303	Protein NRT1/ PTR FAMILY 6.3

Supplemental table 7

DEGs between ER and FER involving in cell wall synthesis

Unigene	Base Mean Exp.		log2	SWISSPROT Description
	ER	FER	FC	
CL30Contig5	0.0	8.7	+∞	Subtilisin-like protease SBT1.6
comp46715_c0_seq2_6	129.8	327.6	1.3	Subtilisin-like protease SBT1.6
CL25Contig11	164.9	390.6	1.2	Subtilisin-like protease SBT1.6
comp50282_c0_seq1_2	11.8	73.2	2.6	Subtilisin-like protease SBT1.7
CL214Contig1	6.3	28.9	2.2	Subtilisin-like protease SBT1.7
CL98Contig9	98.4	352.7	1.8	Subtilisin-like protease SBT1.7
CL421Contig4	209.6	712.6	1.8	Subtilisin-like protease SBT1.7
comp51697_c0_seq1_4	864.2	2719.9	1.7	Subtilisin-like protease SBT1.7
comp41093_c0_seq3_3	56.3	161.3	1.5	Subtilisin-like protease SBT1.7
CL30Contig6	181.7	437.0	1.3	Subtilisin-like protease SBT1.7
CL14148Contig1	54.6	150.6	1.5	Subtilisin-like protease SBT3.3
CL24734Contig1	53.0	184.9	1.8	Subtilisin-like protease SBT4.14
CL3965Contig1	25.7	69.8	1.4	Subtilisin-like protease SBT5.3
CL1322Contig1	28.4	75.4	1.4	Subtilisin-like protease SBT5.4
CL1435Contig2	12.3	56.3	2.2	Probable pectinesterase/pectinesterase inhibitor 40 PME40
comp39057_c0_seq2_3	28.8	81.5	1.5	Probable pectinesterase/pectinesterase inhibitor 51 PME51
comp47585_c0_seq1_5	68.9	192.2	1.5	Probable pectinesterase/pectinesterase inhibitor 51 PME51
CL2301Contig1	26.2	72.7	1.5	Probable pectinesterase/pectinesterase inhibitor 12 PME12
CL2838Contig1	32.6	182.2	2.5	Putative pectinesterase/pectinesterase inhibitor 22 PME22
CL10481Contig1	4.0	23.7	2.6	Pectinesterase/pectinesterase inhibitor 3 PME3
CL8697Contig1	30.8	79.3	1.4	Pectinesterase/pectinesterase inhibitor PPE8B
CL6567Contig1	0.0	27.5	Inf	Leucine-rich repeat extensin-like protein 4
CL11969Contig1	1.0	33.7	5.1	Leucine-rich repeat extensin-like protein 4
CL3391Contig2	80.3	196.1	1.3	Leucine-rich repeat extensin-like protein 2 LRX2
CL1078Contig3	0.3	12.1	5.2	Cellulose synthase-like protein D1
comp38335_c0_seq1_5	37.5	127.8	1.8	Endochitinase
CL692Contig3	56.9	191.2	1.7	Endochitinase
CL10712Contig1	3.7	122.0	5.1	Snakin-1
comp40950_c0_seq1_4	1.0	28.7	4.9	Laccase-4 IRX12
CL4311Contig1	7.5	47.9	2.7	Laccase-4 IRX12
CL8822Contig1	3.0	48.9	4.0	Protein IRX15-LIKE RX15-L
comp51611_c1_seq1_5	1.3	19.8	3.9	Glycerophosphodiester phosphodiesterase GDPDL4
CL24391Contig1	1.0	14.1	3.8	Chaperone protein ClpC1, chloroplastic
CL1078Contig2	1.3	18.0	3.8	Cellulose synthase-like protein D1
comp48655_c0_seq1_2	1.3	17.8	3.8	Glucan endo-1,3-beta-glucosidase 11
CL1601Contig2	58.8	589.2	3.3	Probable xyloglucan endotransglucosylase/hydrolase protein 33
CL5553Contig1	2.3	18.7	3.0	Probable xyloglucan endotransglucosylase/hydrolase protein 10
CL67Contig9	123.5	998.0	3.0	Probable galacturonosyltransferase 4
CL2876Contig1	34.1	257.2	2.9	Sucrose synthase 6
CL5360Contig1	3.3	25.0	2.9	Putative cell wall protein

CL4763Contig1	7.3	47.3	2.7	Endoglucanase 24
CL9113Contig1	3.3	20.9	2.7	Alpha-galactosidase 2
CL8178Contig1	3.6	22.5	2.6	NAC domain-containing protein 73
CL2662Contig1	9.8	61.1	2.6	Pathogenesis-related protein 5
comp51952_c2_seq1_2	12.5	70.1	2.5	Pathogenesis-related protein 5
CL6868Contig1	54.7	305.1	2.5	Monocopper oxidase-like protein SKU5
CL39357Contig1	6.5	36.0	2.5	Probable glycosyltransferase At5g03795
CL653Contig2	15.4	83.7	2.4	Genome polyprotein
CL11226Contig1	261.4	1354.9	2.4	pEARLI1-like lipid transfer protein 2
comp37427_c0_seq1_3	28.8	148.6	2.4	Fasciclin-like arabinogalactan protein 8 FLA8
CL26418Contig1	10.0	50.7	2.3	Glucan endo-1,3-beta-glucosidase 13
CL1198Contig2	9.9	41.2	2.1	Glucan endo-1,3-beta-glucosidase 14
CL3551Contig1	27.7	113.3	2.0	Beta-galactosidase 5 BGAL5
CL16786Contig1	32.4	94.5	1.5	Beta-galactosidase 3 BGAL3
comp39843_c0_seq4_4	31.1	119.9	1.9	Beta-galactosidase 3 BGAL3
CL1648Contig3	55.4	147.6	1.4	Beta-galactosidase 6 BGAL6
CL18158Contig1	150.5	608.3	2.0	Non-classical arabinogalactan protein 31 AGP31
CL37964Contig1	10.8	39.4	1.9	Protein PMR5
CL7794Contig1	110.6	393.0	1.8	Peroxidase 64
comp39821_c0_seq2_1	19.2	67.4	1.8	Probable polygalacturonase non-catalytic subunit JP650
comp14585_c0_seq1_5	110.2	385.3	1.8	Probable polygalacturonase non-catalytic subunit JP650
CL179Contig2	696.8	2412.2	1.8	Probable galacturonosyltransferase-like 10 GATL10
CL5Contig9	91.0	297.7	1.7	Omega-hydroxypalmitate O-feruloyl transferase
CL189Contig1	46.7	151.7	1.7	Endoglucanase 17
CL16784Contig1	37.2	119.5	1.7	Chitinase 8
CL12636Contig1	35.6	107.1	1.6	Glucan endo-1,3-beta-glucosidase 13
comp7326_c0_seq1_4	34.4	103.5	1.6	Endoglucanase 24
CL179Contig4	136.9	411.5	1.6	Probable galacturonosyltransferase-like 3 GATL3
comp48927_c3_seq1_6	412.8	1230.0	1.6	UDP-glycosyltransferase 74B1 UGT74B1
CL4018Contig1	362.7	1038.2	1.5	Osmotin-like protein
CL24316Contig1	46.9	132.8	1.5	GDSL esterase/lipase
CL294Contig1	36.2	100.3	1.5	Pathogenesis-related protein 5
comp37672_c0_seq1_4	1173.8	3229.1	1.5	Aquaporin TIP2-1
comp36435_c0_seq1_6	140.1	376.5	1.4	Aquaporin TIP2-1
CL7445Contig1	106.2	290.8	1.5	Peroxidase 47
CL4859Contig2	89.5	242.5	1.4	Formin-like protein 6
CL24271Contig1	37.9	95.3	1.3	Endoglucanase 8 CEL1
CL6779Contig1	28.6	71.2	1.3	Endochitinase CH5B
CL1483Contig1	337.1	835.7	1.3	Glucan endo-1,3-beta-glucosidase 7
CL4759Contig1	277.2	665.7	1.3	Dirigent protein 21 DIR21
CL5571Contig2	122.5	278.7	1.2	CO(2)-response secreted protease
CL142Contig2	211.2	451.0	1.1	Xyloglucan endotransglucosylase/hydrolase protein 9 XTH9
CL12993Contig1	513.0	1065.5	1.1	Beta-glucosidase 44 BGLU44
CL38813Contig1	86.4	175.7	1.0	Kinesin-like protein BC2

CL6041Contig1	162.8	80.5	-1.0	Germin-like protein subfamily T member 2
CL36Contig2	277.6	116.8	-1.2	Transcription factor MYB26
CL36Contig9	752.0	208.9	-1.8	Transcription factor MYB26
CL3511Contig1	59.0	14.0	-2.1	Transcription factor MYB26
comp52842_c0_seq1_5	522.9	215.4	-1.3	Polygalacturonase
comp48380_c1_seq1_1	729.7	271.1	-1.4	Glucomannan 4-beta-mannosyltransferase 9 CSLA9
CL969Contig2	1587.2	534.7	-1.6	Glucomannan 4-beta-mannosyltransferase 9 CSLA9
CL31245Contig1	4139.7	1498.1	-1.5	Snakin-2
CL1179Contig3	121.7	23.3	-2.4	Cellulose synthase-like protein E6 CSLE6
CL24541Contig1	16.4	1.7	-3.3	Casparian strip membrane protein 1
CL5349Contig1	40.7	4.1	-3.3	D-alanine--D-alanine ligase
CL28037Contig1	15.4	1.3	-3.5	Glucan endo-1,3-alpha-glucosidase agn1
CL17702Contig1	97.5	7.9	-3.6	1,3-beta-glucanosyltransferase gel1
CL25785Contig1	23.9	1.9	-3.7	Calnexin homolog
CL2955Contig2	15.2	1.0	-4.0	Protein trichome birefringence-like 2 TBL2
comp39476_c0_seq1_2	26.4	1.3	-4.4	Fasciclin-like arabinogalactan protein 12 FLA12
CL6438Contig1	98.9	4.2	-4.6	Pectinesterase 2
CL13555Contig1	53.5	1.2	-5.4	Protein ecm33
CL36057Contig1	18.1	0.3	-5.9	14-3-3 protein homolog
CL10910Contig1	14.0	0.0	-∞	Guanine nucleotide-binding protein subunit beta-like protein

Supplemental table 8

RT-qPCR verified unigenes

Targets	Unigene ID	UNIPROT Description	RT-qPCR Primers
Phloem1	CL1969Contig2	Myb family transcription factor APL	AAGAGGGAAACACACAAGAAG<br>ATTCAAGTCCATTTGGACCTAC
Phloem2	CL28929Contig1	Protein DA1-related 2	CTATTCTTGCCCATGAGTTGAT<br>CACATTTGGGAAAGCACCTGA
Vascul1	CL153Contig1	Ethylene-responsive transcription factor 109	GGTAGCAGTAGTGAGATTGTTG<br>GGAATCGGATGAATTGTCGC
Vascul2	CL16199Contig1	WUSCHEL-related homeobox 4	AGGCAAGAACGTGTTCTAC<br>TGGTGTCCAACGTCGTTAT
Vascul3	CL1822Contig1	Ethylene-responsive transcription factor 1A	ACCCTCTCTCTTCTGTCCTA<br>CCCGATCTGTCCATACATT
Translate1	CL24312Contig1	40S ribosomal protein S30-B	GTCGAATCCATCGAACAACAG<br>GACTTGACCTTACCGGCAC
Translate2	CL26328Contig1	Heat shock protein SSB1	TCCGTATCATCAACGAGCC<br>GACATCGAAAGTTCCTCCAC
MYB26	CL3511Contig1	Transcription factor MYB26	AATCGGAGACAAACTCACAG<br>ATGGTTTAGGCAACATGGGTA
Reference	CL15414Contig1	Ubiquitin-conjugating enzyme E2-17 kDa	GTTGAAGGTTCGTTCGAGT<br>TCAAAGGAACCTGTCCAACAC
