# Supplementary material for: Comparative transcriptomics of stem bark reveals genes associated with bast fiber development in Boehmeria nivea L. gaud (ramie)
Source: BMC Genomics. 2020 Jan 13;21:40. doi: 10.1186/s12864-020-6457-8 (PMC6958601; doi:10.1186/s12864-020-6457-8)
Supplement: Supplementary file 2 — Additional file 2: Figure S1. GO analysis of the DEGs between TB and ER. Figure S2. GO analysis of the DEGs between TB and FER. Figure S3. RT-qPCR detection of the selected DEGs among TB, ER and FER. [file 12864_2020_6457_MOESM2_ESM.pdf]

Figure S1

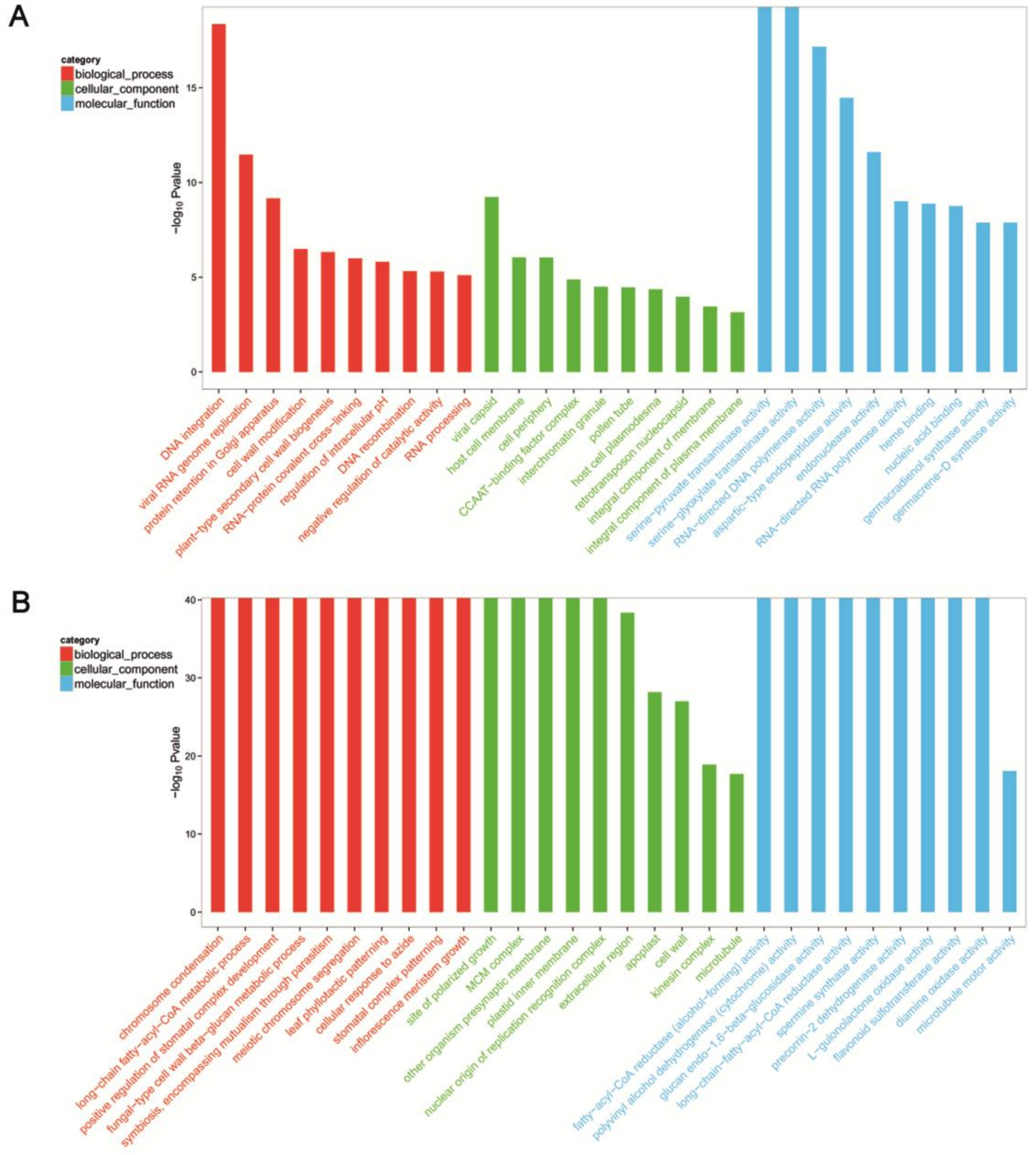

Figure S1 GO analysis of the DEGs between TB and ER. A. Up-regulated DEGs in ER. B. Down-regulated DEGs in ER.

Figure S2

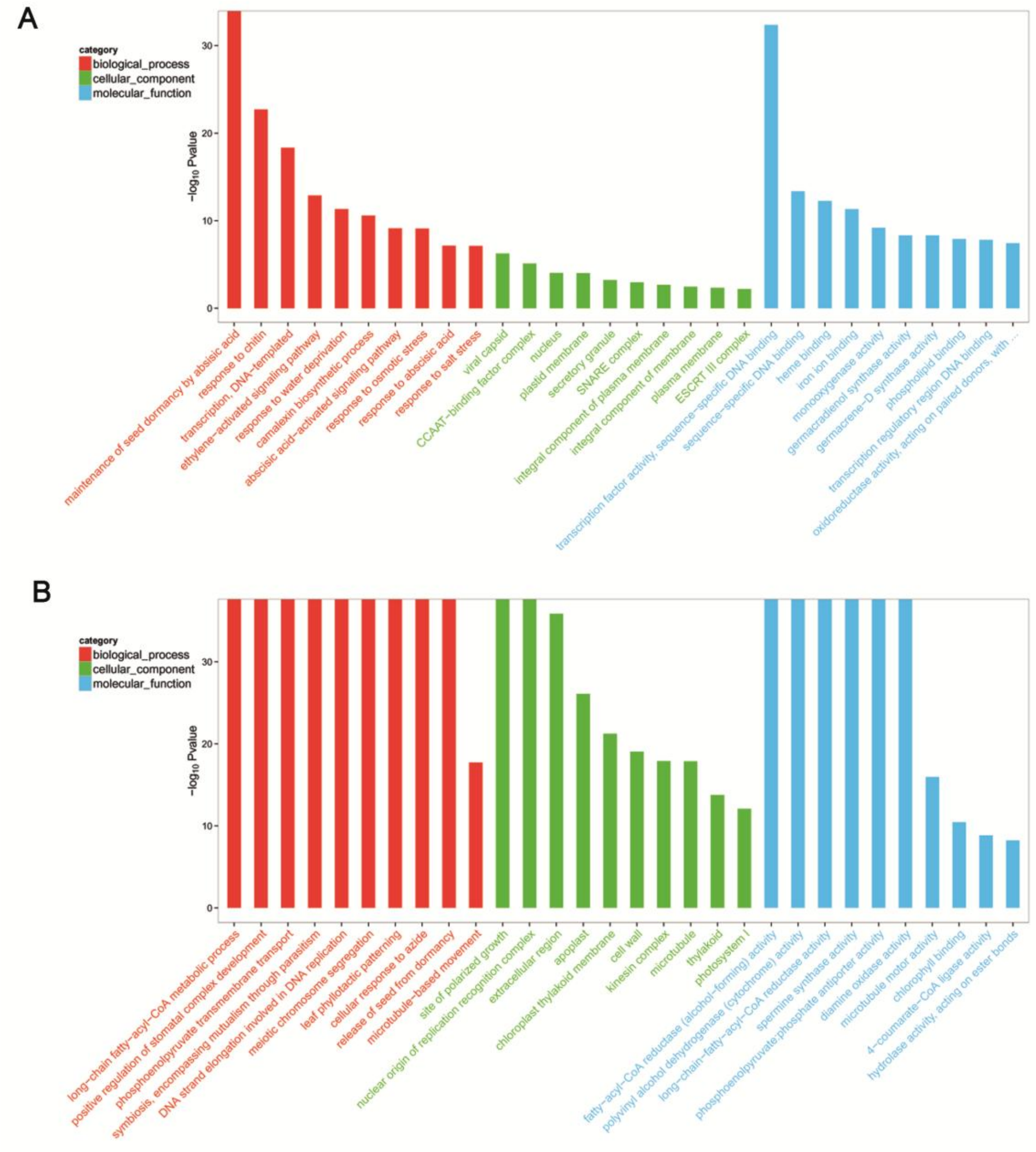

Figure S2 GO analysis of the DEGs between TB and FER. A. Up-regulated DEGs in FER. B. Down-regulated DEGs in FER.

Figure S3

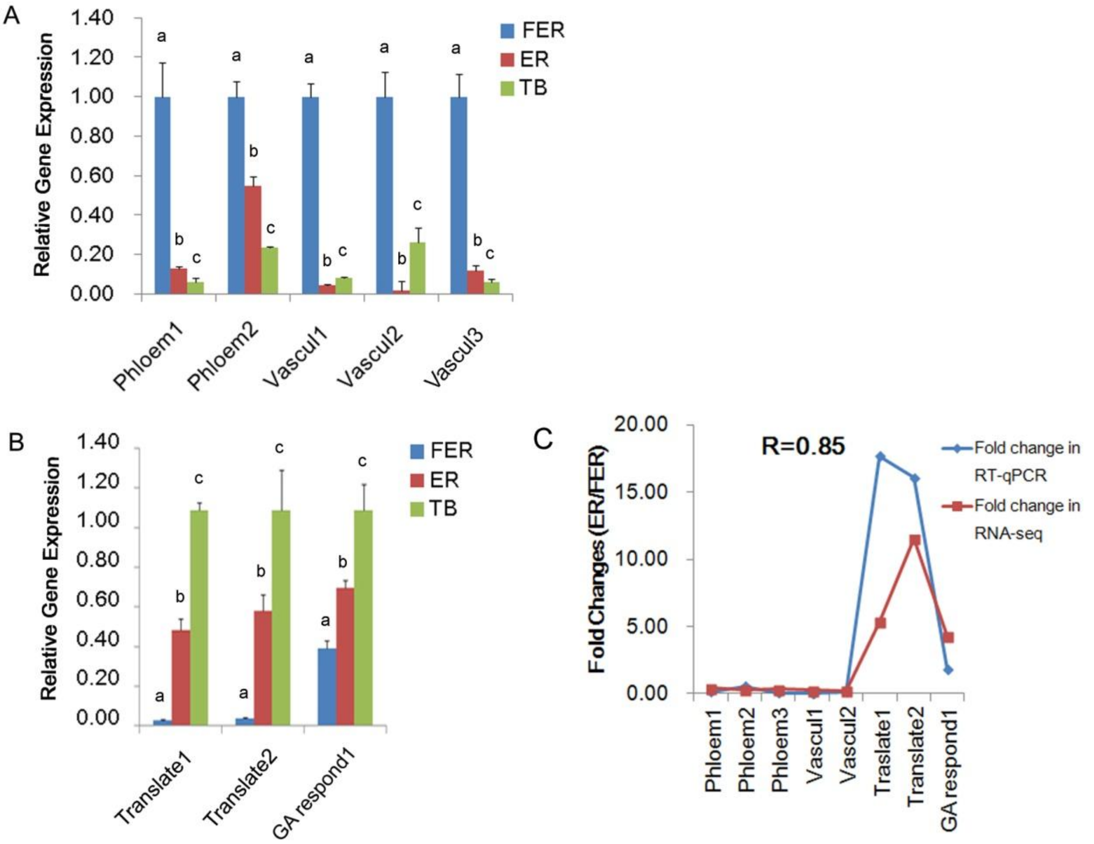

Figure S3 RT-qPCR detection of the selected DEGs among TB, ER and FER. A. Increased expression levels of some of the phloem and vasculature development related DEGs in the FER sample. B. Decreased expression levels of some of the translation and GA responsive factors in the FER sample. Error bars represent the value ±SD (n=3). The lowercase letters above the columns indicate the differences among samples by significance test. C. Fold changes of DEG in both RT-qPCR and RNA-seq FPKM data. The correlation coefficient R=0.85.
